# Growth Enhancement of Arabidopsis (*Arabidopsis thaliana*) and Onion (*Allium cepa*) With Inoculation of Three Newly Identified Mineral-Solubilizing Fungi in the Genus *Aspergillus* Section *Nigri*

**DOI:** 10.3389/fmicb.2021.705896

**Published:** 2021-08-12

**Authors:** Surapong Khuna, Nakarin Suwannarach, Jaturong Kumla, Jens Christian Frisvad, Kenji Matsui, Wipornpan Nuangmek, Saisamorn Lumyong

**Affiliations:** ^1^Research Center of Microbial Diversity and Sustainable Utilization, Faculty of Science, Chiang Mai University, Chiang Mai, Thailand; ^2^Department of Biology, Faculty of Science, Chiang Mai University, Chiang Mai, Thailand; ^3^Department of Biotechnology and Biomedicine, DTU-Bioengineering, Technical University of Denmark, Kongens Lyngby, Denmark; ^4^Graduate School of Sciences and Technology for Innovation, Yamaguchi University, Yamaguchi, Japan; ^5^Faculty of Agriculture and Natural Resources, University of Phayao, Phayao, Thailand; ^6^Academy of Science, The Royal Society of Thailand, Bangkok, Thailand

**Keywords:** black aspergilli fungi, mineral solubilization, plant growth promotion, soil fungi, taxonomy

## Abstract

Some soil fungi play an important role in supplying elements to plants by the solubilizing of insoluble minerals in the soil. The present study was conducted to isolate the mineral-solubilizing fungi from rhizosphere soil in some agricultural areas in northern Thailand. Seven fungal strains were obtained and identified using a polyphasic taxonomic approach with multilocus phylogenetic and phenotypic (morphology and extrolite profile) analyses. All obtained fungal strains were newly identified in the genus *Aspergillus* section *Nigri*, *Aspergillus chiangmaiensis* (SDBR-CMUI4 and SDBR-CMU15), *Aspergillus pseudopiperis* (SDBR-CMUI1 and SDBR-CMUI7), and *Aspergillus pseudotubingensis* (SDBR-CMUO2, SDBR-CMUO8, and SDBR-CMU20). All fungal strains were able to solubilize the insoluble mineral form of calcium, copper, cobalt, iron, manganese, magnesium, zinc, phosphorus, feldspar, and kaolin in the agar plate assay. Consequently, the highest phosphate solubilization strains (SDBR-CMUI1, SDBR-CMUI4, and SDBR-CMUO2) of each fungal species were selected for evaluation of their plant growth enhancement ability on Arabidopsis and onion in laboratory and greenhouse experiments, respectively. Plant disease symptoms were not found in any treatment of fungal inoculation and control. All selected fungal strains significantly increased the leaf number, leaf length, dried biomass of shoot and root, chlorophyll content, and cellular inorganic phosphate content in both Arabidopsis and onion plants under supplementation with insoluble mineral phosphate. Additionally, the inoculation of selected fungal strains also improved the yield and quercetin content of onion bulb. Thus, the selected strains reveal the potential in plant growth promotion agents that can be applied as a biofertilizer in the future.

## Introduction

Elements in soil are important for both plant development and growth (Fernández et al., 2007; El-Ramady et al., 2014). The essential elements for plant growth can be divided into two main groups including macrominerals (calcium, magnesium, nitrogen, phosphorus, potassium, and sulfur) and microminerals (boron, chloride, copper, iron, manganese, molybdenum, nickel, and zinc) (Soetan et al., 2010). Approximately, 95–99% of elements in soil are typically found within insoluble mineral forms such as the carbonate form, phosphate form, oxide form, and complex form (Lian et al., 2008; Gadd, 2010). Thus, soluble elements that are available for plant uptake were found in only 1 to 5% of the soil samples. Insoluble mineral forms of various elements have low availability in most agricultural soil and are, therefore, slowly available for plant uptake (Martínez-Viveros et al., 2010; Haro and Benito, 2019). Plants cultivated in soil that contains those insoluble minerals may be associated with slow degrees of growth and development, as well as with diminished levels of crop productivity. Therefore, chemical fertilizers are commonly being used to increase the amounts of available elements in the soil for plant growth and crop production (Khan et al., 2010). Nevertheless, chemical fertilizers can negatively impact the environment in the way of soil acidification, soil compaction, a reduction in soil fertility and microbial diversity, and heavy metal pollution. Furthermore, the use of chemical fertilizers results in increases in the cost of crop production and can be hazardous to the health of farmers (Gyaneshwar et al., 2002; Etesami et al., 2017; Lin et al., 2019; Hasnain et al., 2020). Currently, many researchers have contributed to the discovery and development of using beneficial microorganisms to partially replace chemical fertilizers, especially certain mineral-solubilizing microorganisms including actinomycetes, bacteria, fungi, and yeasts that can be utilized to establish agricultural sustainability, offer a range of environmental friendly benefits, and restore soil fertility (Pradhan et al., 2017; Hasnain et al., 2020; Kumla et al., 2020; Qarni et al., 2021).

Mineral-solubilizing fungi are a group of beneficial microorganisms that are ubiquitous in the soil. Their presence could play an important role in supplying the necessary soluble elements for plant uptake (Gyaneshwar et al., 2002; Sharma et al., 2013). Previous studies have indicated that mineral-solubilizing fungi make up about 0.1–0.5% of total soil fungal populations (Kucey, 1983; Sharma et al., 2013). Generally, the fungal genera *Aspergillus* and *Penicillium* are the dominant mineral-solubilizing fungi present in soil (Wakelin et al., 2004; Sharma et al., 2013). Previous studies have reported that mineral-solubilizing fungi have the potential to solubilize the insoluble form of minerals making them soluble for uptake in plants (Alori et al., 2017; Sattar et al., 2019). Moreover, they can also improve plant growth through the production of phytohormones, antibiotics, and siderophores while helping to control plant diseases (Mittal et al., 2008; Sharma et al., 2013). Several previous studies reported that the inoculation of mineral-solubilizing fungi in the genera *Aspergillus*, *Fomitopsis*, *Penicillium*, *Talaromyces*, and *Trichoderma* has enhanced plant growth and crop yields for several plants (Arabidopsis, chickpeas, chili, Chinese cabbage, haricot beans, mung beans, onions, and tomatoes) while also increasing soil fertility (Manjunath et al., 1981; Yadav et al., 2011; Jain et al., 2012a; Wang et al., 2015; Elias et al., 2016; Zhao et al., 2017, 2018; Naziya et al., 2019). Thus, the inoculation of mineral-solubilizing fungi is a promising strategy that can be employed to increase available elements for uptake in plants and reduce the need for chemical fertilizers (Sharma et al., 2013; Alori et al., 2017; Abdel-Motaal et al., 2020). This study aimed to isolate the mineral-solubilizing fungi from agricultural areas in Chiang Mai Province in the north of Thailand. The obtained mineral-solubilizing fungi were identified through morphological characteristics, extrolite profiles, and multigene phylogenetic analysis. All obtained fungal strains were evaluated for their capability in the solubilization of insoluble metal minerals. Subsequently, some obtained fungal strains were selected for evaluation in terms of their ability to enhance the growth of Arabidopsis and onion in both laboratory and greenhouse experiments, respectively.

## Materials and Methods

### Isolation of Mineral Solubilizing Fungi

Soil samples were collected from three sites located inside a longan orchard of Mae Wang District, Chiang Mai Province, Northern Thailand in August 2017. Soil samples were dried at room temperature (25 ± 2°C) in the dark for 5 days. Then, soil samples were ground and sieved with a mash to 2 mm prior to the isolation of the fungi. The isolation of mineral-solubilizing fungi followed the methodology described by Parmar and Sindhu (2013) with some modifications. The serial dilution spread plate technique was prepared using a 0.5% (w/v) NaCl solution with three serial dilutions. After that, 0.1 ml of the suspension was plated on modified Aleksandrov agar. The isolation plates were then incubated at 30°C for 5 days in the dark. Fungal colonies with a halo zone around colonies indicated the mineral-solubilizing strains. Pure fungal colonies were purified by a single hyphal tip method described by Korhonen and Hintikka (1980) on potato dextrose agar (PDA; CONDA, Spain) and were used for further experiments. Each pure fungal strain was kept on PDA slants at 4°C for short-term preservation and in 20% glycerol at −20°C for long-term preservation.

### Identification of Mineral Solubilizing Fungi

#### Morphological Studies

Colony characteristics on nine agar media, including PDA, Czapek agar (CZA; Difco, France), Czapek yeast extract agar (CYA; sucrose 30.0 g, yeast extract 5.0 g, K_2_HPO_4_ 1.0 g, KCl 0.5 g, NaNO_3_ 3.0 g, MgSO_4_⋅7H_2_O 0.5 g, FeSO_4_⋅7H_2_O 0.01 g, agar 15.0 g, in 1 L of deionized water, and pH 6.2), CYA supplemented with 5% NaCl (CYAS), malt extract agar (MEA; Difco, France), oatmeal agar (OA; Difco, France), yeast extract sucrose agar (YES; sucrose 20 g, yeast extract 4.0 g, MgSO_4_ 0.5 g, KH_2_PO_4_ 1.0 g, agar 15.0 g, and in 1 L of deionized water), creatine sucrose agar (CREA; creatine 3.0 g, sucrose 30 g, K_2_HPO_4_⋅3H_2_O 1.3 g, FeSO_4_⋅7H_2_O 0.5 g, KCl 0.5 g, MgSO_4_⋅7H_2_O 0.5 g, bromocresol purple 0.05 g, agar 15.0 g, in 1 L of deionized water, and pH 8.0) was determined in this study. Colony diameter and macromorphological characters on each agar medium were observed after incubation at 25 and 37°C in the dark for 1 week. Three replicates of each agar medium were made. Micromorphological characteristics were performed under light microscope (Nikon ECLIPSE E200, Tokyo, Japan) according to the method described by previous studies (Samson et al., 2004, 2007, 2014; Silva et al., 2011). Size data of the anatomical properties, e.g., conidia heads, stipes, vesicles, metulae, phialides, and conidia, were measured with at least 50 numbers of each structure.

For scanning electron microscopy (SEM), conidia were obtained from CZA following the method of Fungaro et al. (2017). A mycelial plug (5 × 5 mm) was fixed using 2% (v/v) of glutaraldehyde in 0.1 M NaPO_4_ buffer at 4°C for 24 h. The plug was washed every 15 min, three times, in 0.1 M NaPO_4_ buffer and was then postfixed in 1% (w/v) OsO_4_ buffer at 25°C in the dark for 2 h. The plug was then washed again at 15 min, three times, in 0.1 M NaPO_4_ buffer and dehydrated in 70, 80, 90, and 100% ethanol series for 10 min of each concentration. Then, the plug was placed in a critical point dryer and subsequently sputter-coated with gold. The samples were assessed and photographed a using scanning electron microscope (JEOL JSM-5910 LV, Japan).

#### DNA Extraction, PCR Amplification, and Sequencing

Genomic DNA of each fungal strain was extracted from mycelia grown on CZA in the darkness for 5 days by FAVOGEN DNA Extraction Mini Kit (FAVOGEN, Taiwan). Five genes were amplified using polymerase chain reaction (PCR). The calmodulin (*cam*), β-tubulin (*benA*), RNA polymerase II second largest subunit (*rpb2*), actin (*act*), and translation elongation factor 1-α (*tef1*) genes were amplified with primer pairs CF1/CF4 (Peterson, 2008), Bt2a/Bt2b (Glass and Donaldson, 1995), bRPB2-6F/bRPB2-7.1R (Matheny, 2005), A-TEF_F/A-TEF_R (Perrone et al., 2011), and ACT-512F/ACT-783R (Carbone and Kohn, 1999), respectively. The amplification of five genes was conducted in separate PCR reactions. The amplification process consisted of an initial denaturation at 95°C for 3 min, followed by 35 cycles of 30-s denaturation at 95°C, annealing of 1 min at 51°C (*cam*); 30 s at 52°C (*benA* and *act*); 30 s at 59°C (*tef1*) and 1 min at 54°C (*rpb2*), and 1 min extension at 72°C; followed by a final extension at 72°C for 10 min. PCR products were purified using NucleoSpin Gel and a PCR Clean-up Kit (Macherey-Nagel, Germany) according to the procedure of the manufacturer and then sent to a commercial service provider (1st Base Company, Kembangan, Malaysia) for sequencing. The obtained sequences were used to query the BLASTN search in the GenBank database^1^.

#### Sequence Alignment and Phylogenetic Analyses

The details of sequences used for phylogenetic analyses are presented in Supplementary Table 1. The multiple sequence alignments were performed using MUSCLE (Edgar, 2004). The combined *cam*, *benA*, *rpb2*, *tef1*, and *act* alignment was submitted in TreeBASE under the study ID 27054. The phylogenetic tree was determined by maximum likelihood (ML) and Bayesian inference (BI) algorithms implemented by RAxML v. 7.0.3 and MrBayes v3.3.6, respectively (Stamatakis, 2006; Ronquist et al., 2012). The best substitution models for BI and ML analyses were approximated using jModeltest 2.1.10 (Darriba et al., 2012) by Akaike information criterion (AIC). Both ML and BI analyses were based on the GTR + I + G model. *Aspergillus fischeri* CBS 544.65 and *Aspergillus novofumigatus* CBS 117520 within section Fumigati were used as the outgroup. ML analysis was assessed using a bootstrap (BS) with 1,000 replicates (Felsenstein, 1985). The phylogenetic tree was visualized using the program Treeview 32 (Page, 2001). BS values above or equal to 70% were regarded as significantly and strongly supported for clades (Hillis and Bull, 1993). The BI analysis was conducted using the program MrBayes v. 3.2 by the Metropolis-coupled Markov chain Monte Carlo (MCMCMC) method (Ronquist et al., 2012). Markov chains were run for 1 million generations, starting from random trees with six chains. A sampling chains was performed at every 100th generation. Among these, the first 2,000 trees were discarded as burn-in, and the Bayesian posterior probabilities were estimated for the postburn-in trees by constructing the 50% majority-rule consensus phylogram. Bayesian posterior probability (PP) values above or equal to 0.95 were considered to be significantly supported (Alfaro et al., 2003).

### Extrolite Analysis

The fungal strains were grown on CYA and YES for 1 week at 25°C prior to extrolite extraction following the methods described by Nielsen et al. (2011) and Frisvad et al. (2019). Four plugs (5 mm in diameter) of each agar medium were taken and combined in the same vial. Then, 0.75 ml of a mixture of methanol/dichloromethane/ethyl acetate (1:2:3, v/v/v) with 1% (v/v) formic acid was added to the vial. The solvent was evaporated, and then methanol was added into the dried extract mixture. The extract was filtered and 1 μl was injected into an Agilent high-performance liquid chromatograph. Extrolite profile of each fungal strain was identified with an Agilent Infinity 1290 HPLC system (Agilent Technologies, Santa Clara, CA, United States) using high-performance liquid chromatography-diode array detection-high-resolution quadrupole time of flight mass spectrometry (HPLC-DAD-HRqTOFMS) as described in detail by Nielsen et al. (2011) and Kildgaard et al. (2014).

### Evaluation of Ability to Solubilize Insoluble Minerals

Seven fungal strains derived from pure cultures were determined for their ability to solubilize insoluble minerals on agar plates. This experiment was conducted using basal medium with the supplementation of 0.5% (w/v) insoluble minerals that included aluminum phosphate (AlPO_4_), calcium carbonate (CaCO_3_), calcium phosphate [Ca_3_(PO_4_)_2_], cobalt carbonate (CoCO_3_), copper carbonate [CuCO_3_⋅Cu(OH)_2_], copper oxide (CuO), feldspar (KAlSi_3_O_8_), ferric phosphate (FePO_4_), kaolin [Al_2_Si_2_O_5_(OH)_4_], magnesium carbonate (MgCO_3_), manganese oxide (MnO), zinc carbonate (ZnCO_3_), and zinc oxide (ZnO) following the procedure described by Fomina et al. (2005) and Kumla et al. (2014). The medium was sterilized at 121°C for 15 min. After the sterilization process, the test media (25 ml) was poured into sterile Petri dishes. Mycelial plugs (5 mm in diameter) were inoculated into the center of the tested media and incubated at 30°C in the dark for 5 days. Then, fungal colony diameters and halo zones (solubilization zone) were measured. Three replications of each of the treatments were conducted. The ratio of the halo zone diameter to the fungal colony diameter was calculated and expressed as the solubilization index (SI) (Vitorino et al., 2012). Mineral solubilization activities of low, medium, and high were established at less than 1.0, from 1.0 to 2.0, and more than 2.0, respectively.

### Plant Growth Promotion by Selected Mineral Solubilizing Fungi

#### Fungal Inoculum Preparation

The most effective fungal strain of each fungal species recorded from the previous experiment (*Aspergillus chiangmaiensis* SDBR-CMUI4, *Aspergillus pseudopiperis* SDBR-CMUI1, and *Aspergillus pseudotubingensis* SDBR-CMUO2) were selected and used in this study. The selected fungal strain was grown on PDA medium at 37°C for 1 week. Conidia were collected in a Petri dish containing 5 ml of sterilized deionized water by gentle scraping. Conidial concentration of the suspension was performed using a hemocytometer under a microscope (Wang et al., 2015). The carrier material was prepared as a mixture of vermiculite, perlite, and peat moss at a ratio of 5:2:3 (w/w/w). The mixed carrier material was dried at 70°C for 72 h, run through a blender, sieved through a 2-mm mesh, and sterilized by being run twice through an autoclave set at 121°C for 30 min. The sterilized carrier material was mixed with the conidial suspension of each fungal strain at a final concentration of about 1 × 10^7^ conidia/g (Raymond et al., 2018). The granulation process was employed by using a tablet pressure machine. After that, the granules were then dried in an oven at 45°C for 48 h before being used.

### Plant Growth Promotion in Arabidopsis Under Laboratory Conditions

The experiment was carried out in a plant growth room from October to November of 2019. The room was located at the Faculty of Agriculture, Yamaguchi University, Japan. Seeds of *Arabidopsis thaliana* (ecotype Col-0) were surface disinfected for 3 min in 70% ethanol and 12 min in 2% sodium hypochlorite. They were subsequently washed five times using sterile distilled water. The seeds were then dried for 2–3 h in the laminar flow cabinet. In this experiment, soil mixed with vermiculite (3:1, w/w) with pH values in the range of 6.8–7.0 was used as the planting material. The planting material was sterilized at 121°C for 60 min. Surface disinfected seeds were sown in plastic pots (6.5 × 5.5 cm) containing 80 g of sterilized planting material, and the pots were incubated at 4°C in darkness over 2 days. After that, pots were transferred to a growth room (light intensity of 12,680 lx, and 70% relative humidity), and seeds were germinated for 14 days at 22°C for a 16-h photoperiod. This experiment was conducted using a completely randomized design (CRD) with eight separate treatments as indicated in Table 1. To initiate individual growth, seedlings were carefully transferred to the new pots containing 80 g of planting material in each experiment. Ten replications were made for each treatment, and experiments were run twice. Seedlings were incubated in a growth room, and growth parameters were measured according to the method described below.

**TABLE 1:** Treatment details in this study.

Treatment number	Treatment details
T1	Planting material (control)
T2	Planting material (1 kg) + Ca_3_(PO_4_)_2_ (500 mg)
T3	Planting material (1 kg) + inoculum of *Aspergillus chiangmaiensis* SDBR-CMUI4 (1 g)
T4	Planting material (1 kg) + inoculum of *Aspergillus pseudopiperis* SDBR-CMUI1 (1 g)
T5	Planting material (1 kg) + inoculum of *Aspergillus pseudotubingensis* SDBR-CMUO2 (1 g)
T6	Planting material (1 kg) + Ca_3_(PO_4_)_2_ (500 mg) + inoculum of *A. chiangmaiensis* SDBR-CMUI4 (1 g)
T7	Planting material (1 kg) + Ca_3_(PO_4_)_2_ (500 mg) + inoculum of *A. pseudopiperis* SDBR-CMUI1 (1 g)
T8	Planting material (1 kg) + Ca_3_(PO_4_)_2_ (500 mg) + inoculum of *A. pseudotubingensis* SDBR-CMUO2 (1 g)

#### Plant Growth Promotion for Onions Under Greenhouse Conditions

Seeds of onion (*Allium cepa*) cultivar “Super Rex” were surface disinfected for 5 min in 5% sodium hypochlorite, rinsed three times using sterile distilled water, and then soaked in sterile distilled water for 10 h. The seeds that sank to the bottom of the vessel were used for the experiments. Commercial soil (Sompong Kan Kaset Company, Phayao Province, Thailand) with pH values in the range of 6.8–6.9 was used as the planting material in this experiment. The soil was sterilized two times at 121°C for 30 min. Seeds were sown in seedling trays with 72 holes that contained sterilized soil. This experiment was arranged using a completely randomized design (CRD). The details of each treatment in this experiment are described in Table 1. Forty-five days after seed germination, seedlings were transferred into each plastic pot (15 × 11.5 × 10.5 cm) containing 1 kg of planting material in each experiment. Ten replications of each treatment were run twice. Plants were grown for 90 days in a greenhouse located at the School of Agriculture and Natural Resources, University of Phayao, Phayao Province, located in Northern Thailand during the period of November 2017 to February 2018. The maximum temperature and relative humidity in the greenhouse ranged from 20 to 30°C and 65 to 85%, respectively. The maximum daily light intensity was in the range of 13,000 to 35,000 lx.

#### Measurement and Yield of Plant Growth

The total number of leaves, leaf length, and dry weight of the roots were determined for both of Arabidopsis and onion plants. In addition, rosette diameter, length of the main root and the leaf width of Arabidopsis plants were measured and recorded according to the method described by Rahmoune et al. (2017) and Hanlon et al. (2018). Moreover, the height of the plants, the dry weight of the leaves, bulb fresh weight, bulb diameter, bulb height, and bulb quality of the onion plants were measured and recorded by following the method described in previous studies (Amin et al., 2011; Bettoni et al., 2014; Petropoulos et al., 2015).

### Determination of Chlorophyll and Cellular Inorganic Phosphate Contents in Plant

Chlorophyll content (chlorophyll a, chlorophyll b, and total chlorophyll) in leaves of Arabidopsis and onion plants was measured following the method described by Lichtenthaler and Wellburn (1983) and Liang et al. (2017). Fresh leaf samples (0.2 g) were soaked in 8 ml of 80% (v/v) acetone solution and then incubated at 25°C for 24 h in the dark until the tissue turned white. The absorption at 645 and 663 nm was determined with the supernatant. The concentration of chlorophyll was calculated, and data were expressed as mg/g.

Cellular inorganic phosphate content was determined following the method described by Ames (1966) and Wang et al. (2012). Plant tissues were weighed and submerged in 1% glacial acetate (1 ml). They were then frozen and thawed eight times. Subsequently, 200 μl of deionized water and 700 μl of phosphate reaction buffer (A = 0.42% ammonium molybdate, 2.85% (v/v) sulfuric acid, B = 10% (w/v) ascorbic acid, A:B (v/v) in a ratio of 6:1) was mixed with 100-μl volume of the extract. The reaction was incubated at 37°C for 60 min, and the absorbance was determined at 820 nm using a spectrophotometer. The concentration of cellular inorganic phosphate content was calculated from a calibration curve of dipotassium hydrogen phosphate, and data were expressed as μmol/g fresh weight (μmol/g FW).

#### Determination of Chemical Constituents in Onion Bulb

In this study, total soluble solids, titratable acidity, and quercetin content in onion bulbs were determined. Onion juice was extracted from each onion bulb in each treatment following the procedure of Chope et al. (2006). The extract was kept in 15 ml of microcentrifuge tubes and stored at −20°C. Total soluble solids of the extracts were determined following the methodology of Magwaza and Opara (2015). One milliliter of the extract was used and measured with the use of a digital refractometer (Model PAL-1, Japan), and the results were expressed in degrees Brix (°Brix).

Titratable acidity was estimated using the titration method described by Petropoulos et al. (2015). One milliliter of extract was taken in a titration flask, and 9 ml of distilled water was added. The extract was then titrated with 0.1 N NaOH and 1% (w/v) phenolphthalein as an indicator until an endpoint was reached (permanent light pink color appeared), and the titer value was noted. Data were calculated and expressed as the percentage of citric acid (% citric acid).

Quercetin concentration was investigated according to the method of Kwak et al. (2017) with some modifications. Extracts were analyzed using Shimadzu Prominence UFLC system equipped with an LC-20 AD pump, a CTO-20 AC column oven, a SIL-20ACHT autosampler, a CBM-20A system controller, and a SPD-20A UV/VIS detector (Shimadzu, Japan). Mightysil RP-18 GP (150 × 2.0 mm, 5 μm) column was used and set at 40°C. The mobile phase consisted of water:formic acid (95:5, v/v) (A) and 100% methanol (B). The binary gradient that was used in this experiment was prepared as follows: 0–22 min, 20–60% B; 22–22.1 min, 60–100% B; 22.1–25 min, 100–60% B; 25–25.1 min, 60–20% B; 25.1–30 min, 20% B. The injection volume and flow rate were set at 10 μl and 0.3 ml/min, respectively. The detection wavelength was 360 nm. The authentic quercetin standard (Sigma, Germany) with different levels was constructed for a calibration curve. The quercetin concentration was quantified by correlating peak areas of the sample extract and the calibration curve. Results are presented as milligram per gram of dry weight (mg/g DW).

### Statistical Analysis

Statistical differences between treatments were assessed using one-way ANOVA with the SPSS program for Microsoft Windows (version 16; SPSS Inc., United States). Duncan’s multiple range test (DMRT) was used to determine significant differences at *p* ≤ 0.05.

## Results

### Isolation of Mineral Solubilizing Fungi

A total of seven fungal strains (SDBR-CMUI1, SDBR-CMUI4, SDBR-CMUI7, SDBR-CMUO2, SDBR-CMUO8, SDBR-CMU15, and SDBR-CMU20) displayed positive mineral-solubilizing ability by producing a clear zone around the colony on modified Aleksandrov agar. These seven strains were selected for use in further experiments. All fungal strains were deposited at Thailand Bioresource Research Center (TBRC), Pathum Thani Province, and in the Sustainable Development of Biological Resources (SDBR) Laboratory, Faculty of Science, Chiang Mai University, Chiang Mai Province, Thailand.

### Identification of Selected Mineral-Solubilizing Fungi

#### Morphological Observations

Fungal colonies of each strain were observed on different agar media (PDA, CZA, CYA, CYAS, MEA, OA, YES, and CREA) at different temperatures (25 and 37°C), and the results are presented in Table 2. After being incubated for 7 days, CZA was found to be the best media by displaying the highest colony diameter of all seven strains. All seven fungal strains produced conidiophores, vesicles, metulae, phialides, and dark brown conidia in all of the agar media. Based on these morphological observations, all strains were initially identified as belonging to the genus *Aspergillus* section *Nigri* (Abarca et al., 2004; McClenny, 2005; Samson et al., 2007, 2014). Therefore, molecular methods and extrolite profiles were applied to confirm the identification of the obtained fungal strains.

**TABLE 2:** Colony diameter of seven fungal strains in this study on different media at 25 and 37°C after 7 days of incubation in the darkness.

Media	Colony diameter (mm)					
	*A. chiangmaiensis* ^a,b^		*A. pseudopiperis* ^c,d^		*A. pseudotubingensis* ^e,f,g^	
	25°C	37°C	25°C	37°C	25°C	37°C
PDA	68–69	74–76	78–80	83–85	67–70	77–80
CZA	82–83	>85	>85	>85	>85	>85
CYA	62–63	57–66	63–65	61–64	51–58	55–68
CYAS	49–52	50–52	55–57	55–67	46–48	43–48
MEA	68–70	73–76	55–60	60–62	62–63	68–70
OA	63–65	70–72	65–67	76–78	63–65	65–66
YES	63–65	60–63	70–72	75–77	65–67	56–58
CREA	41–42	51–52	46–47	55–57	42–43	53–54

*^a^Strain SDBR-CMUI4, ^b^strain SDBR-CMU15, ^c^strain SDBR-CMUI1, ^d^strain SDBR-CMUI7, ^e^strain SDBR-CMUO2, ^f^strain SDBR-CMUO8, and ^g^strain SDBR-CMU20.*

#### Phylogenetic Results

**FIGURE 1:**
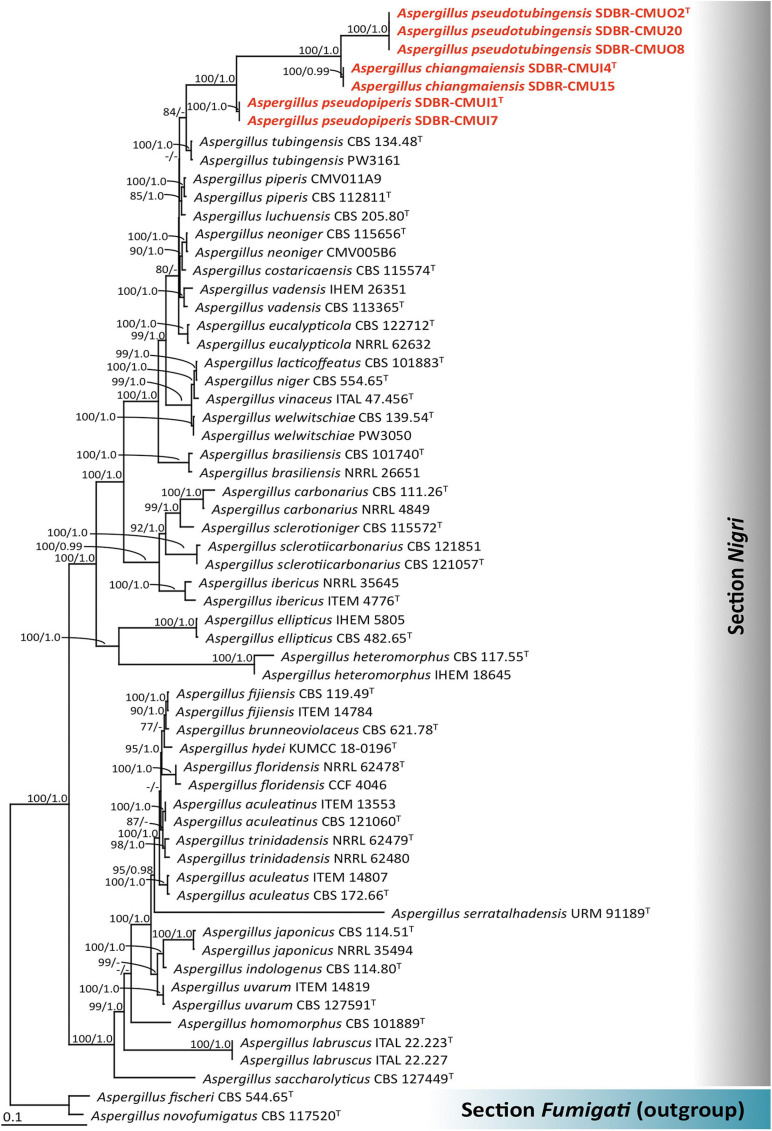
Phylogenetic tree derived from maximum likelihood (ML) analysis of the combined *cam*, *benA*, *rpb2*, *tef1*, and *act* genes of 61 sequences. *Aspergillus fischeri* and *Aspergillus novofumigatus* were used as outgroup. Numbers above branches are bootstrap (BS) values (left) and posterior probability (PP) values (right). Branches with BS and PP values more than 70% and 0.95, respectively, are shown at each branch. Bar represents 0.1 substitutions per nucleotide position. Hyphen (-) represents support values ≤ 70%/0.95. Superscript “T” indicates the type species. The fungal strains obtained in this study are represented in bold red.

The *cam*, *benA*, *rpb2*, *tef1*, and *act* sequences of the seven fungal strains were deposited in GenBank (Supplementary Table 1). In this study, phylogenetic trees were generated using combined data comprised of five genes (*cam*, *benA*, *rpb2*, *tef1*, and *act*) consisting of 61 strains, and the aligned dataset was comprised of 4,624 characters including gaps (*cam*: 1–779, *benA*: 780–1386, *rpb2*: 1387–2573, *tef1*: 2574–3423, and *act*: 3424–4624). ML analysis revealed that the gamma shape parameters for the rates of nucleotide substitution among the variable sites and the proportion of the invariable sites were 0.9480 and 0.2510, respectively. Additionally, the tree with a final log likelihood value of −31,216.5263 was obtained. The average standard deviation of the split frequencies of the BI analysis was 0.006039. The topologies from ML and BI analyses were similar (data not shown). Thus, ML tree was selected and presented. A phylogram of the combined *cam*, *benA*, *rpb2*, *tef1*, and *act* sequences is shown in Figure 1. The results of the combined analysis showed that seven fungal strains obtained in this study was assigned in three monophyletic clades and clearly formed distinct lineages within *Aspergillus* section *Nigri* with high BS and PP supports. Therefore, three new species of *Aspergillus* section *Nigri* were described herein as *A. chiangmaiensis*, *A. pseudopiperis*, and *A. pseudotubingensis*.

### Taxonomy Description

#### *Aspergillus chiangmaiensis* S. Khuna, N. Suwannarach & S. Lumyong, sp. nov. (Figure 2)

MycoBank: MB830887

**FIGURE 2:**
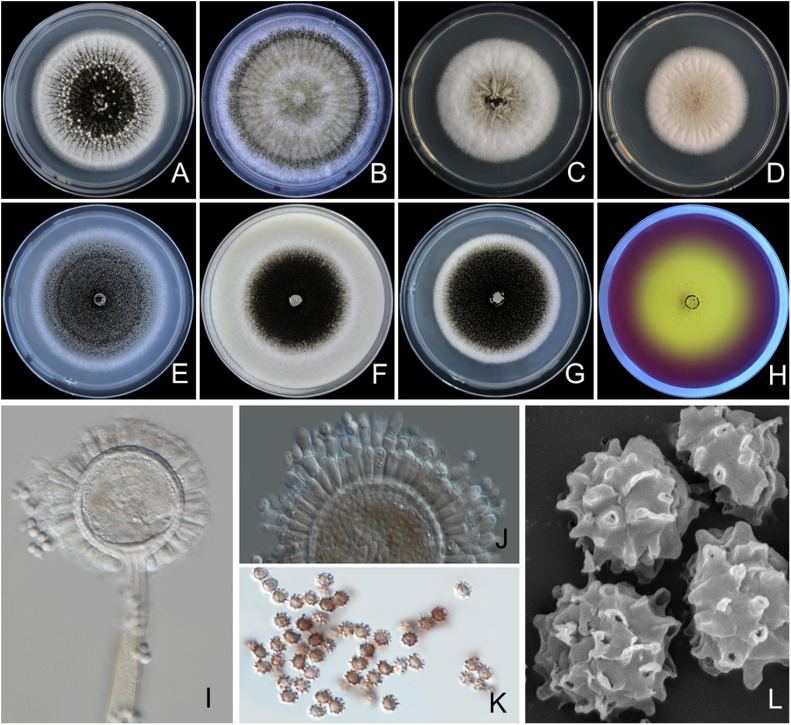
*Aspergillus chiangmaiensis* SDBR-CMUI4 (holotype). Colonies incubated at 25°C for 7 days. **(A)** Potato dextrose agar (PDA), **(B)** Czapek agar (CZA), **(C)** Czapek yeast agar (CYA), **(D)** CYA supplemented with 5% NaCl (CYAS), **(E)** malt extract agar (MEA), **(F)** oatmeal agar (OA), **(G)** yeast extract sucrose agar (YES), **(H)** creatine sucrose agar (CREA), **(I,J)** conidiophores under light microscopy, **(K)** conidia under light microscopy, **(L)** conidia as seen using scanning electron microscopy (SEM). Scale bars: **(A–H)** 10 mm, **(I–K)** 10 μm, and **(L)** 5 μm.

*Etymology*: *chiangmaiensis*, referring to Chiang Mai Province where soil containing the new fungus was collected.

*Holotype*: THAILAND. Chiang Mai Province, Mae Wang District, (18°36′46″N, 98°46′30″E), isolated from the soil of a longan orchard, August 8, 2017, S. Khuna, dried culture: SDBR-CMUI4; ex-type culture: TBRC10407. GeneBank: MK457199 (*cam*), MK457200 (*benA*), MK457202 (*rpb2*), MK457203 (*tef1*), and MK457201 (*act*).

*Culture characteristics*: The colonies first are white, floccose, then conidiophores are abundantly produced, conidial areas are dark brown to black on PDA, CZA, CYA, CYAS, MEA, OA, YES, and CREA (Figures 2A–H). Reverse cream to light brown on PDA, CZA, and YES; cream to light yellow on CYA, CYAS, and OA; light brown to black on MEA. On CREA, thin colonies with poor sporulation, and strong acid production. Conidiophores are produced abundantly on OA and YES. Sclerotia are present on PDA and CZA, white, globose to ellipsoidal, 390–1,375 × 390–1,085 μm (Figures 2A,B). Conidial heads are globose, dark brown, radiate, commonly splitting into columns with age, 60–225 μm in diameter. Conidiophores are biseriate with globose to ellipsoidal vesicles 35–68 μm (Figures 2I,J), stipes are smooth, thick walled, 370–1,430 × 10–15-μm wide near the vesicle, light brown. Metulae covering virtually the entire surface of the vesicle, 14–25 × 3–5 μm. Phialides ampulliform, 8–12 × 2–3 μm (Figure 2J). Conidia globose to subglobose, 3–4.5 μm in diameter, dark brown, with an echinulate surface (Figures 2K,L).

*Extrolite production*: Desertorin C, pyrophen, secalonic acid D, and a unique ustilaginoidin-like compound.

*Additional specimens examined*: THAILAND, Chiang Mai Province, Mae Wang District, (18°36′46″N, 98°46′30″E), isolated from the soil of a longan orchard, August 8, 2017, S. Khuna, SDBR-CMU15.

*Notes*: *A. chiangmaiensis* can be distinguished from other current accepted species of *Aspergillus* section *Nigri* in that it produces globose to ellipsoidal white sclerotia on CZA and PDA CZA at 25°C and 37°C (Supplementary Table 2). The characteristics of the colonies of *A*. *chiangmaiensis* were similar to *A. ibericus*, but the latter species did not produce sclerotia. In addition, the growth of *A. chiangmaiensis* displayed faster growth than *A. ibericus* (38–43 mm) on CZA at 25°C (Serra et al., 2006). The microscopic characteristics and size of the biseriate species in *Aspergillus* section *Nigri* were compared, and the findings are shown in Supplementary Table 3. Based on the micromorphological characteristics, both species, *A*. *chiangmaiensis* and *A. ibericus*, are biseriate species. However, *A. ibericus* differs from *A*. *chiangmaiensis* by its larger conidial heads (500–600 μm), metulae (30–40 × 5.0–7.5 μm), phialides (8–10 × 6–7 μm), and conidia (5–7 μm) (Serra et al., 2006). In addition, *A. chiangmaiensis* produced echinulate conidia, but *A. ibericus* produced conspicuously verruculose with spines projecting conidia (Serra et al., 2006). A phylogenetic tree revealed that *A. chiangmaiensis* formed distinct lineages within the *Aspergillus* section *Nigri* and is sister to *A. pseudotubingensis* with high support (100% BS, 1.0 PP; Figure 1). *A. pseudotubingensis* differ from *A. chiangmaiensis* by the absence of sclerotia on any culture medium and presents finely spinose conidia and its extrolite production. Extrolite profiles: *A. chiangmaiensis* is similar to *A. ibericus* in the way that both do not produce ochratoxin A (Serra et al., 2006), but *A. chiangmaiensis* could produce desertorin C, pyrophen, secalonic acid D, and a unique ustilaginoidin-like compound (Supplementary Table 4).

#### *Aspergillus pseudopiperis* S. Khuna, N. Suwannarach & S. Lumyong, sp. nov. (Figure 3)

MycoBank: MB830888

**FIGURE 3:**
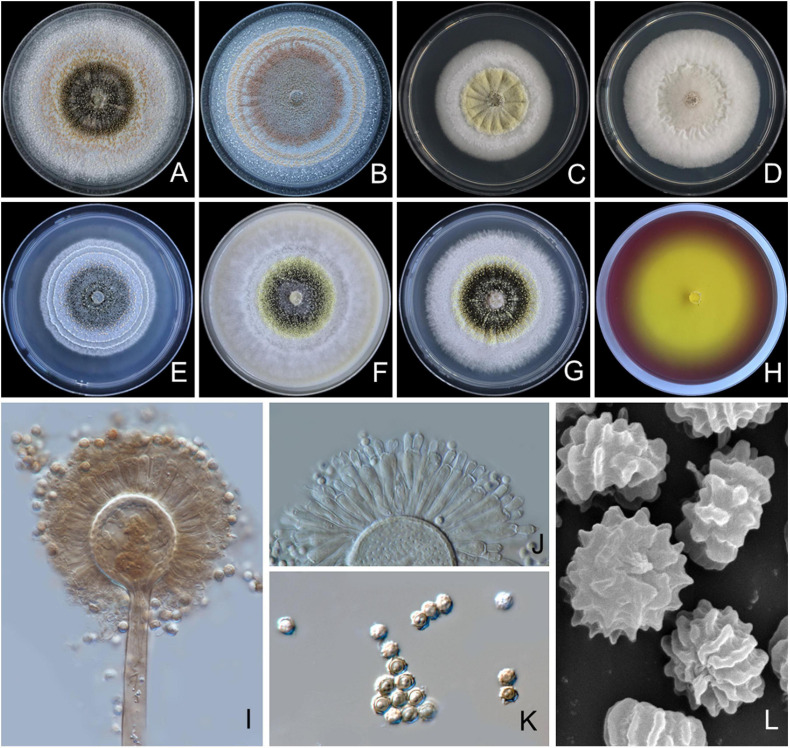
*Aspergillus pseudopiperis* SDBR-CMUI1 (holotype). Colonies incubated at 25°C for 7 days. **(A)** PDA, **(B)** CZA, **(C)** CYA, **(D)** CYAS, **(E)** MEA, **(F)** OA, **(G)** YES, **(H)** CREA, **(I,J)** conidiophores under light microscopy, **(K)** conidia under light microscopy, **(L)** conidia as seen using SEM. Scale bars: **(A–H)** 10 mm, **(I–K)** 10 μm, and **(L)** 5 μm.

*Etymology*: *pseudo* = false, referring to the colony characteristics that are easily mistaken for *A. piperis*.

*Holotype*: THAILAND. Chiang Mai Province, Mae Wang District, (18°36′46″N, 98°46′30″E), isolated from the soil of a longan orchard, August 8, 2017, S. Khuna, dried culture: SDBR-CMUI1; ex-type culture: TBRC10408. GeneBank: MK457193 (*cam*), MK457194 (*benA*), MK457196 (*rpb2*), MK457197 (*tef1*), and MK457195 (*act*).

*Culture characteristics*: The colonies, first white, floccose, then conidiophores, are sparsely produced, conidial areas are greenish brown to dark brown on PDA, CZA, CYA, CYAS, CY20S, MEA, OA, YES, and CREA (Figures 3A–H). Reverse cream to light brown on PDA, CZA, MEA and YES; cream to light yellow on CYA, CYAS, and OA. On CREA, thin colonies with poor sporulation and strong acid production. Conidiophores are abundant on PDA and YES. Sclerotia are present on PDA, CZA, CYA, MEA, OA, and YES, light yellow to pinkish orange, globose to ellipsoidal, 200–985 × 185–765 μm. Conidial heads are globose, dark brown, radiate, commonly splitting into columns with age, 50–245 μm in diameter. Conidiophore is biseriate with globose to ellipsoidal vesicles 15–55 μm (Figures 3I–J), stipes smooth, thick walled, 200–2,125 × 10–18 μm wide near vesicle, light brown. Metulae covering virtually the entire surface of the vesicle, 15–39 × 3–7 μm. Phialides ampulliform, 9–14 × 3–4 μm (Figure 3J). Conidia globose to subglobose, 3–5 μm in diameter, dark brown, with ridge surface (Figures 3K,L).

*Extrolite production*: Aflavinines, emindole, rotiorin, and a unique ustilaginoidin-like compound.

*Additional specimens examined*: THAILAND, Chiang Mai Province, Mae Wang District, (18°36′46″N, 98°46′30″E), isolated from the soil of a longan orchard, August 8, 2017, S. Khuna, SDBR-CMUI7.

*Notes*: *A. pseudopiperis* can be distinguished from other species of *Aspergillus* section *Nigri* by its light yellow to pinkish orange sclerotia on CYA, CZA, MEA, OA, PDA, and YES, with the exception of *A. piperis* (Samson et al., 2004) (Supplementary Table 2). The smaller sclerotia of *A. pseudopiperis* (200–985 × 185–765 μm) was clearly different from *A. piperis* (1,000–1,700 μm) (Samson et al., 2004). In addition, the growth of *A. pseudopiperis* on YES at 25°C was slower than *A. piperis* (79–83 mm), but *A. pseudopiperis* displayed faster growth than *A. piperis* (45–54 mm) on OA at 25°C (Samson et al., 2004). Based on the micromorphological characteristics, both species, *A. pseudopiperis* and *A. piperis* are biseriate species. However, *A. pseudopiperis* can be distinguished from *A. piperis* by its longer phialides. *A. pseudopiperis* produced ridged conidia, but *A. piperis* produced smooth when young to very rough with irregular bar/striation conidia (Samson et al., 2004). A phylogenetic tree revealed that *A. pseudopiperis* formed distinct lineages within the *Aspergillus* section *Nigri* and is sister to *A. pseudotubingensis* and *A. chiangmaiensis* with high support (100% BS, 1.0 PP; Figure 1). *A. pseudotubingensis* differ from *A. pseudopiperis* by its production of finely spinose conidia, while *A. chiangmaiensis* presents echinulate conidia (Supplementary Table 3). Additionally, the extrolite profiles of *A. pseudopiperis* differ from *A. pseudotubingensis* and *A. chiangmaiensis* by its production of aflavinines, emindole, and rotiorin (Supplementary Table 4).

#### *Aspergillus pseudotubingensis* S. Khuna, N. Suwannarach & S. Lumyong, sp. nov. (Figure 4)

MycoBank: MB830889

**FIGURE 4:**
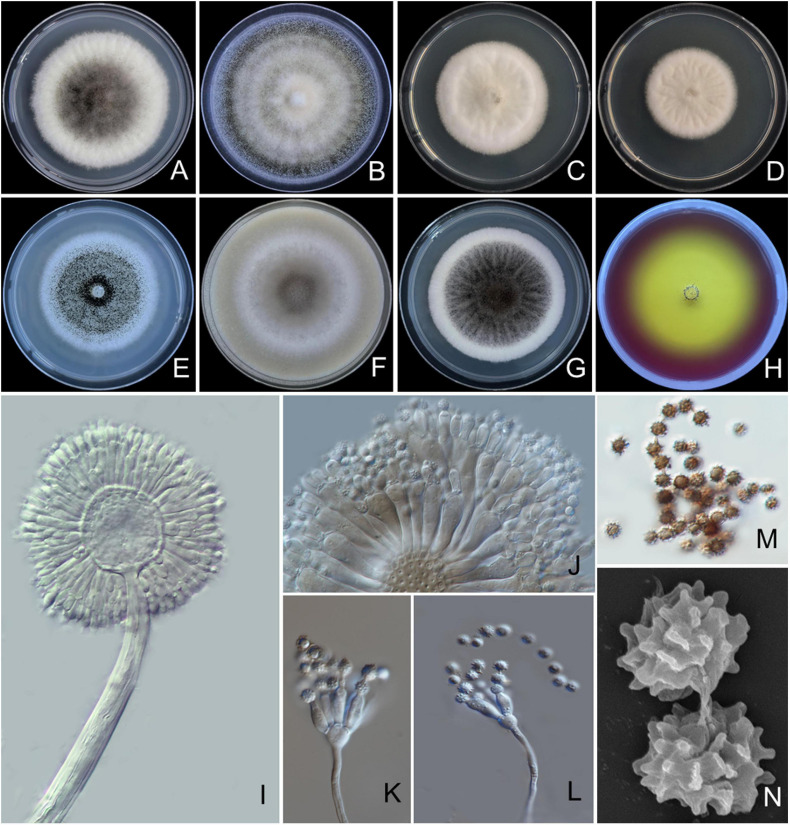
*Aspergillus pseudotubingensis* SDBR-CMUO2 (holotype). Colonies incubated at 25°C for 7 days. **(A)** PDA, **(B)** CZA, **(C)** CYA, **(D)** CYAS, **(E)** MEA, **(F)** OA, **(G)** YES, **(H)** CREA, **(I–L)** conidiophores under light microscopy, **(M)** conidia under light microscopy, **(N)** conidia as seen using SEM. Scale bars: **(A–H)** 10 mm, **(I–M)** 10 μm, and **(N)** 5 μm.

*Etymology*: *pseudo* = false, referring to the colony characteristics that are easily mistaken for *A. tubingensis*.

*Holotype*: THAILAND. Chiang Mai Province, Mae Wang District (18°36′46″N, 98°46′30″E), isolated from the soil of a longan orchard, August 8, 2017, S. Khuna, dried culture: SDBR-CMUO2; ex-type culture: TBRC10409. GeneBank: MK457205 (*cam*), MK457206 (*benA*), MK457208 (*rpb2*), MK457209 (*tef-1*), and MK457207 (*act*).

*Culture characteristics*: The colonies, first white, floccose, then conidiophores, are abundantly produced, conidial areas are grayish brown to dark brown on PDA, CZA, CYA, CYAS, MEA, OA, YES, and CREA (Figures 4A–H). Reverse cream to light yellow on PDA, CZA, CYA, CYAS, OA, and YES; pale on MEA. On CREA, thin colonies with poor sporulation and strong acid production. Conidiophores are abundant on PDA and YES. On PDA, CZA, CYA, MEA, OA, and YES. No sclerotia were observed in all agar media. Conidial heads are globose, dark brown, radiate, commonly splitting into columns with age, 50–255 μm in diameter. Conidiophore biseriate with globose to ellipsoidal vesicles 20–70 μm (Figures 4I–L), stipes smooth, thick walled, 740–3,060 × 10–18 μm wide near vesicle, light brown. Metulae covering virtually the entire surface of the vesicle, 14–46 × 3–6 μm. Phialides ampulliform, 9–14 × 3–4 μm (Figure 4J). Conidia globose to subglobose, 3–6 μm in diameter, dark brown, with fine spiny surface (Figures 4M,N).

*Extrolite production*: Brasenol and tensidol C.

*Additional specimens examined*: THAILAND, Chiang Mai Province, Mae Wang District, (18°36′46″N, 98°46′30″E), isolated from the soil of a longan orchard, August 8, 2017, S. Khuna, SDBR-CMUO8, and SDBR-CMU20.

*Notes*: *A. pseudotubingensis* can be distinguished from other species of *Aspergillus* section *Nigri* by no sclerotium formation on any culture medium. The colony characteristics of *A*. *pseudotubingensis* were similar to *A. tubingensis*. However, the growth of *A. pseudotubingensis* displayed faster growth than *A. tubingensis* (35–45 mm) on CZA at 25°C (Horn et al., 2013). Based on the micromorphological characteristics, both species, *A*. *pseudotubingensis* and *A. tubingensis*, are biseriate species. *A. pseudotubingensis* revealed longer metulae than *A. tubingensis*. *Aspergillus tubingensis* produced tuberculate to aculeate with ridges conidia, but *A. pseudotubingensis* produced fine spiny conidia (Horn et al., 2013). A phylogenetic tree showed that *A*. *pseudotubingensis* formed distinct lineages within the *Aspergillus* section *Nigri* and is sister to *A. chiangmaiensis* with high support (100% BS, 1.0 PP; Figure 1). *A. chiangmaiensis* differ from *A*. *pseudotubingensis* by it presents echinulate conidia and its extrolite profile (Supplementary Tables 3, 4). *A. pseudotubingensis* differs from *A. tubingensis* and other biseriate species by producing brasenol and tensidol C (Samson et al., 2004, 2007; de Vries et al., 2005; Serra et al., 2006; Varga et al., 2007, 2011; Noonim et al., 2008; Frisvad et al., 2011; Hong et al., 2013) (Supplementary Table 4).

### Insoluble Mineral Solubilization Ability

**FIGURE 5:**
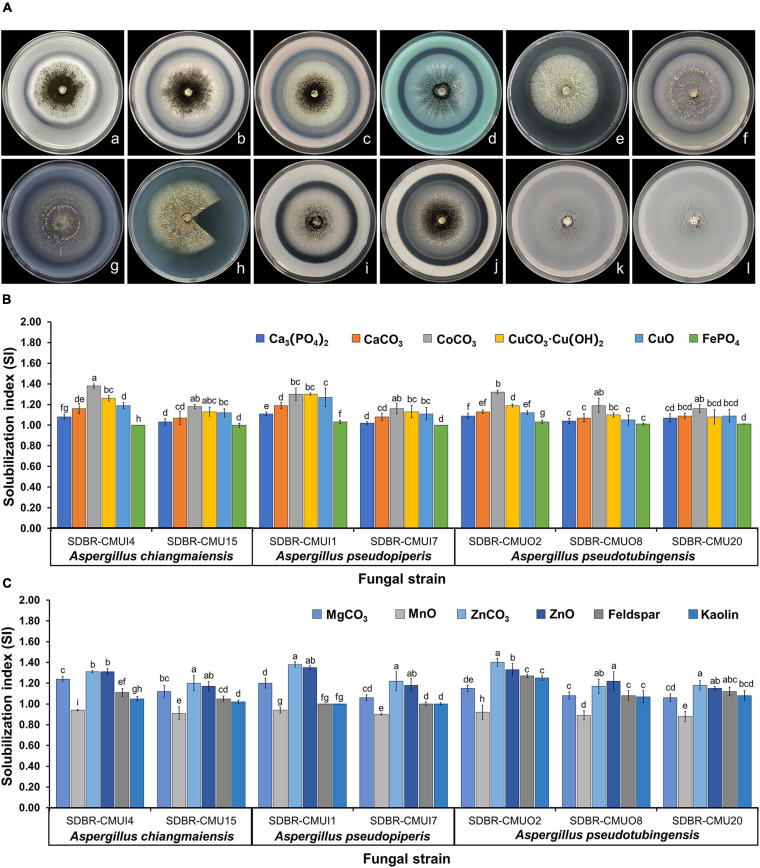
Solubilization of insoluble minerals in agar media by the mineral-solubilizing fungi **(A)** and solubilization index of the ability to solve insoluble minerals **(B,C)**. Error bars represent the standard deviation of the mean. The different letters indicate significant difference (*p* ≤ 0.05). *A. chiangmaiensis* SDBR-CMUI4 on basal medium contained of Ca_3_(PO_4_)_2_ **(a)**, CaCO_3_ **(b)**, CoCO_3_ **(c)**, CuCO_3_⋅Cu(OH)_2_ **(d)**; *A. pseudopiperis* SDBR-CMUI1 on basal medium contained CuO **(e)**, FePO_4_ **(f)**, MgCO_3_ **(g)**, MnO **(h)**; *A. pseudotubingensis* SDBR-CMUO2 on basal medium contained ZnCO_3_ **(i)**, ZnO **(j)**, feldspar **(k)**, kaolin **(l)**. Scale bars: **(a–l)** 10 mm. Fungal colony in **h** was cut for observing a solubilization area (halo zone) beneath the fungal colonies.

Seven fungal strains (*A. chiangmaiensis* SDBR-CMUI4 and SDBR-CMU15; *A*. *pseudopiperis* SDBR-CMUI1 and SDBR-CMUI7; and *A*. *pseudotubingensis* SDBR-CMUO2, SDBR-CMUO8, and SDBR-CMU20) were used in this experiment. Their ability to solubilize insoluble minerals depended on the type of minerals and fungal strains. For the solubilization activities, both solubilization zones (halo zone) that are larger and beneath the fungal colonies were observed (Figure 5A). The results showed that all fungal strains could solubilize all tested insoluble metal minerals, except Al_3_(PO_4_)_2_. The SI value was calculated for solubilization activity and is shown in Figures 5B,C. It was found that the solubilization activity of all fungal strains in the presence of cobalt, copper, ferric, magnesium, manganese, phosphorus, zinc-containing minerals, feldspar, and kaolin was characterized as medium activity (1.0 < SI < 2.0). The low solubilization activity of all fungal strains was found in MnO (SI < 1.0).

### Plant Growth Promotion in Arabidopsis Under Laboratory Conditions

**FIGURE 6:**
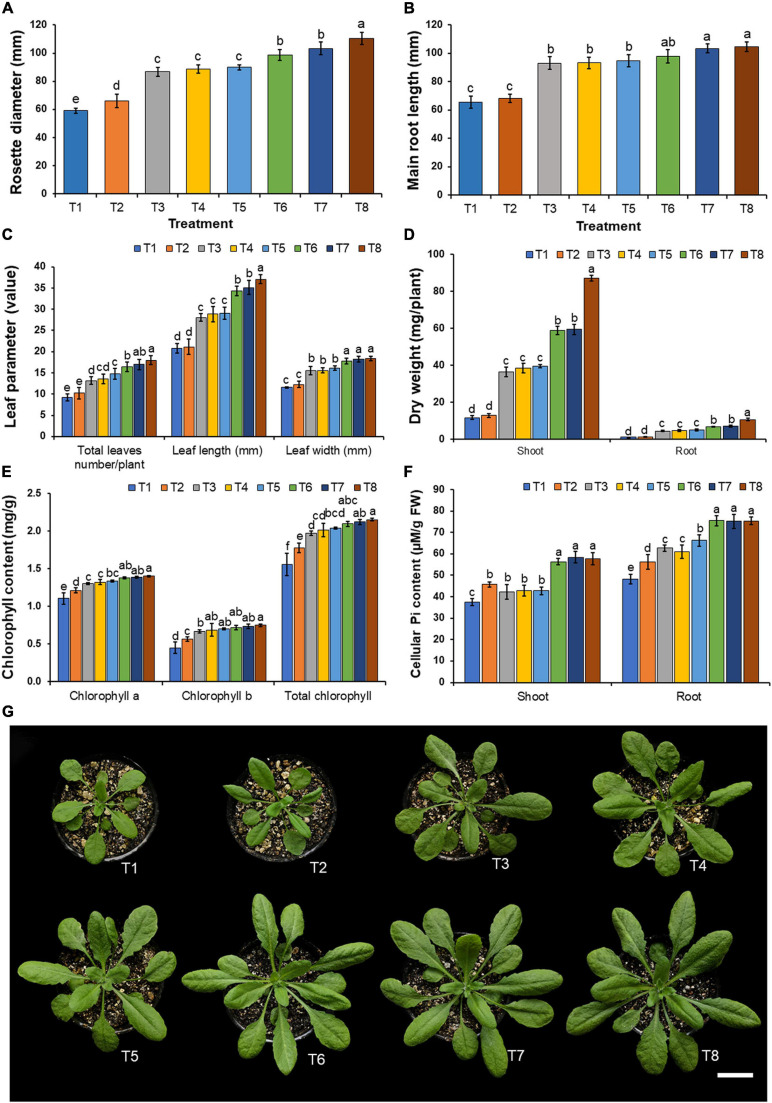
Effect of mineral-solubilizing fungi on the growth of Arabidopsis. **(A)** Rosette diameter, **(B)** main root length, **(C)** total leaf number, leaf length and leaf width, **(D)** shoot and root dry weight, **(E)** chlorophyll content, **(F)** cellular inorganic phosphate content, **(G)** Arabidopsis in each treatment. Error bars represent the standard deviation of the mean. The different letters indicate significant difference (*p* ≤ 0.05). Scale bar: **(G)** 2 cm.

Plant disease symptoms were not observed in any treatment of fungal inoculation and in the control during the planting period. The results indicate that Arabidopsis plants inoculated with each mineral-solubilizing fungus supplemented with the insoluble mineral phosphate (T6–T8) were found to have been significantly increased in terms of rosette diameter, main root length, the number of leaves, and leaf length and width when compared with the plants in other treatments after 21 days of planting period (Figures 6A–C,G). It was found that the growth of Arabidopsis plants inoculated with each mineral-solubilizing fungus (T3–T5) was higher than the Arabidopsis plants in the control treatment (T1). The highest values of dried shoot and root weight were obtained in Arabidopsis plants that had been inoculated with *A. pseudotubingensis* SDBR-CMUO2 (T8) in the plants supplemented with the insoluble mineral phosphate, followed by Arabidopsis plants inoculated with each *A. chiangmaiensis* SDBR-CMUI4 (T6) and *A. pseudopiperis* SDBR-CMUI1 (T7) in the plants supplemented with the insoluble mineral phosphate, respectively (Figure 6D). However, the lowest dry weight value of the shoots and roots was found in the control treatment (T1).

The results indicated that the supplementation of only insoluble mineral phosphate (T2) and the inoculation of each mineral-solubilizing fungus in both non-supplemented (T3–T5) and supplemented treatments involving insoluble mineral phosphate (T6–T8) increased the chlorophyll content in the leaves and cellular inorganic phosphate content in the shoots and roots of Arabidopsis plants when compared with plants in the control treatment (T1) (Figures 6E,F). The highest value of chlorophyll content in the leaves was found in Arabidopsis plants inoculated with *A. pseudotubingensis* SDBR-CMUO2 (T8) under conditions supplemented with the insoluble mineral phosphate. Cellular inorganic phosphate content values in the shoots and roots of Arabidopsis plants in all specimens inoculated with mineral-solubilizing fungi under conditions supplemented with insoluble mineral phosphate (T6–T8) were significantly higher than in the other treatments. The lowest contents of chlorophyll and the cellular inorganic phosphate were found in Arabidopsis plants of the control treatment (T1).

### Plant Growth Promotion in Onions Grown Under Greenhouse Conditions

**FIGURE 7:**
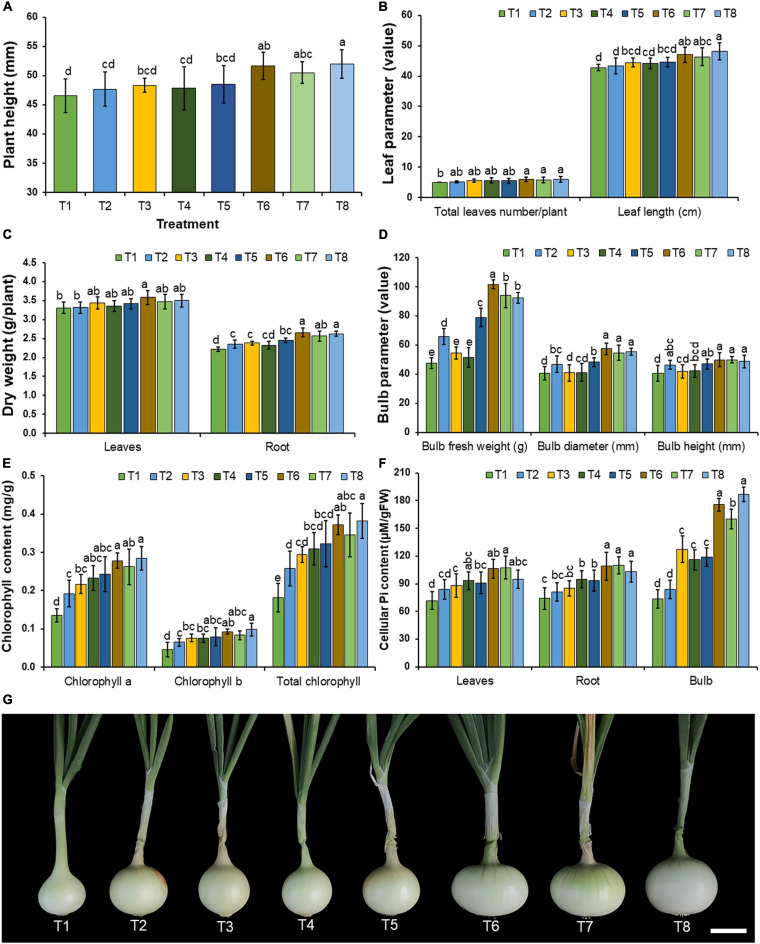
Effect of mineral-solubilizing fungi on the growth and yield of onion. **(A)** Plant height, **(B)** total leaf number and leaf length, **(C)** leaves and root dry weight, **(D)** bulb fresh weight, bulb diameter, and bulb height, **(E)** chlorophyll content, **(F)** cellular inorganic phosphate content, **(G)** onion bulb in each treatment. Error bars represent the standard deviation of the mean. The different letters indicate significant difference (*p* ≤ 0.05). Scale bar: **(G)** 2 cm.

During the planting period, plant disease symptoms were not observed in any treatment of fungal inoculation and in the control. The results indicated that the supplementation of only insoluble mineral phosphate (T2) and the inoculation of each mineral-solubilizing fungus in the experiments under conditions involving both the non-supplementation (T3–T5) and supplementation of insoluble mineral phosphate (T6–T8) could increase the growth of onions when compared with the control treatment (T1) after 90 days of planting (Figures 7A,B,G). It was found that the inoculation of mineral-solubilizing fungi under conditions supplemented with insoluble mineral phosphate (T6–T8) could significantly improve the root dry weight, bulb fresh weight, and bulb diameter of onions when compared with the other treatments (Figures 7C,D).

Chlorophyll content in the leaves and cellular inorganic phosphate content in the leaves, roots, and bulbs of onions are shown in Figures 7E,F, respectively. The results indicated that all inoculations of mineral-solubilizing fungi under conditions supplemented with the insoluble mineral phosphate (T6–T8) significantly increased the cellular inorganic phosphate content in the roots and bulbs of onions when compared with the other treatments.

**FIGURE 8:**
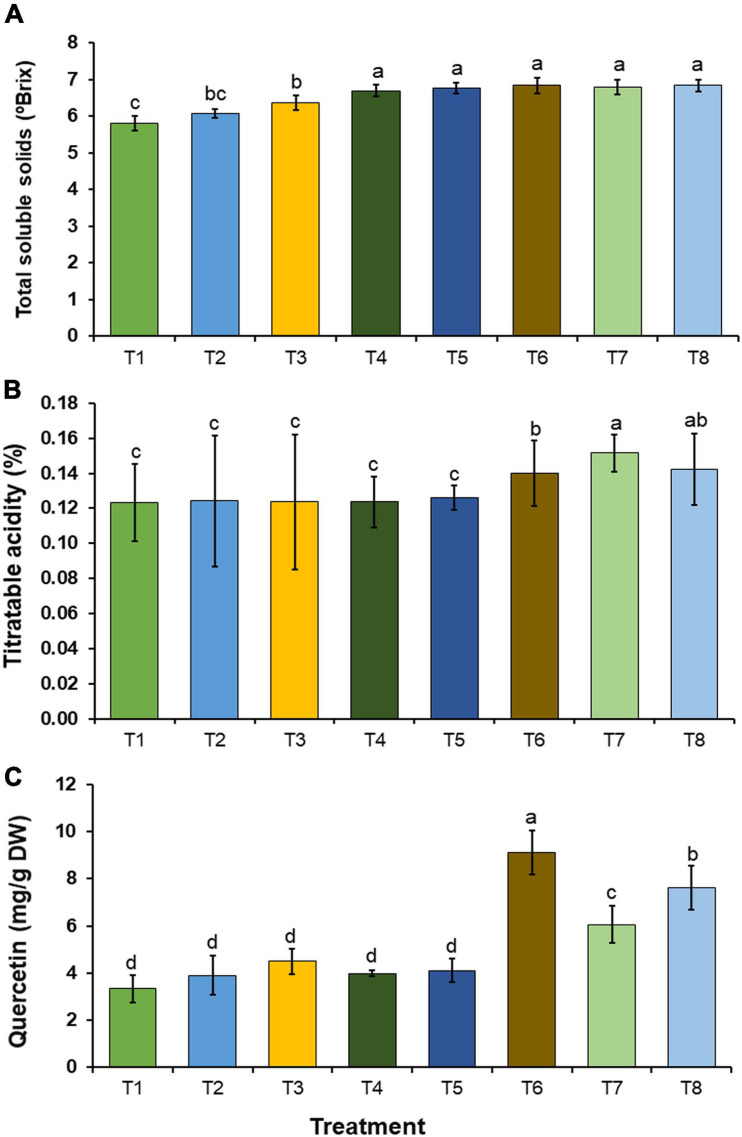
Chemical constituents in onion bulb. **(A)** Total soluble solids, **(B)** titratable acidity, **(C)** quercetin content. Error bars represent the standard deviation of the mean. The different letters indicate significant difference (*p* ≤ 0.05).

Total soluble solids, titratable acidity, and quercetin content in the onion bulbs in each treatment were investigated, and the results are shown in Figure 8. Quercetin content was determined using HPLC. The results indicated that the quercetin content in the onion bulbs corresponded to the quercetin standard with a retention time of 18.4 min (Supplementary Figure 1). The amount of quercetin in the onion bulbs was also quantified by HPLC. In this study, the obtained values of total soluble solids, titratable acidity, and quercetin content in the onion bulbs were within the ranges of 5.80–6.83°Brix, 0.123–0.152%, and 3.34–9.11 mg/g DW, respectively. The inoculation of mineral-solubilizing fungi under conditions of both the non-supplementation (T3–T5) and supplementation of insoluble mineral phosphate (T6–T8) significantly increased total soluble solids in onion bulbs when compared with onion bulbs in control treatment (T1) (Figure 8A). Interestingly, all inoculations of mineral-solubilizing fungi under conditions supplemented with the insoluble mineral phosphate (T6–T8) were found to have significantly increased titratable acidity and quercetin content in onion bulbs when compared with the other treatments (Figures 8B,C).

## Discussion

In the present study, three new mineral-solubilizing species of *Aspergillus* section *Nigri* were isolated from the soil of a longan orchard in northern Thailand. The identification was based on a polyphasic approach, with a combination of molecular data, morphology, physiology, and extrolite data. This approach was also used for species delimitation in the genus *Aspergillus* by the previous studies (Crous et al., 2018; Vesth et al., 2018; Matsuzawa et al., 2019; Cai et al., 2020) to clarify the taxonomic position of *Aspergillus* species. Previous studies have reported that *benA* and *cam* gene regions are required for validation of the *Aspergillus* species, while *rbp2*, *tef1*, and *act* genes can be used in *Aspergillus* taxonomy (Geiser et al., 2007; Perrone et al., 2008; Jurjević et al., 2012; Fungaro et al., 2017; Crous et al., 2018; Vesth et al., 2018; Matsuzawa et al., 2019; Cai et al., 2020). Our phylogenetic analyses of a combined five genes (*cam*, *benA*, *rpb2*, *tef1*, and *act*) revealed that three new species, *A. chiangmaiensis*, *A. pseudopiperis*, and *A. pseudotubingensis*, formed distinct lineages within the *Aspergillus* section *Nigri*. Morphologically, *A*. *chiangmaiensis*, *A. pseudopiperis*, and *A. pseudotubingensis* have been distinguished from the currently accepted species of *Aspergillus* section *Nigri* (Samson et al., 2014; Fungaro et al., 2017; Crous et al., 2018; da Silva et al., 2020; Doilom et al., 2020). The different morphological characteristics identified between the three new species have indicated that only *A. chiangmaiensis* and *A. pseudopiperis* could produce sclerotia (Supplementary Table 2). Additionally, the micromorphological characteristics indicated that *A. chiangmaiensis* presents echinulate conidia, while *A. pseudopiperis* presents ridged conidia, and *A. pseudotubingensis* presents finely spinose conidia (Supplementary Table 3). Furthermore, extrolite profiles have been shown to be useful for species differentiation of the *Aspergillus* species section *Nigri* (Samson et al., 2007; Varga et al., 2007; Perrone et al., 2008; Nielsen et al., 2009; Silva et al., 2011). The extrolite profiles of three new species, *A. chiangmaiensis*, *A. pseudopiperis*, and *A. pseudotubingensis* differed, and their extrolite profiles also differed from the other biseriate species of *Aspergillus* section *Nigri* (Supplementary Table 4). However, Pyrophen and secalonic acid D production by *A. chiangmaiensis* were found in *A. brasiliensis* (Varga et al., 2007) and *A*. *homomorphus* (Samson et al., 2007), respectively. Aflavinines were produced by *A. pseudopiperis*, and these indoloterpenes are produced in most sclerotia of black *Aspergillus* species (TePaske et al., 1989; Samson et al., 2007; Frisvad et al., 2014).

Soil fungi have been known for the important microorganisms that are involved in the biogeochemical cycling of elements in the terrestrial environment (Gadd, 2010, 2017; Fra̧c et al., 2018). Several previous studies have determined that soil fungi in the genera *Aspergillus*, *Arthroderma*, *Apophysomyces*, *Fusarium*, *Geotrichum*, *Mucor*, *Penicillium*, *Rhizopus*, *Talaromyces*, *Trichoderma*, and *Tritirachium* could effectively solubilize various insoluble minerals (Akintokun et al., 2007; Acevedo et al., 2014; Kanse et al., 2015; Patel et al., 2015; Yin et al., 2015; Zhao and Zhang, 2015; Gaind, 2016; Kasana et al., 2017; Karmakar et al., 2018; Khuna et al., 2019). In this study, all pure fungal strains of *A. chiangmaiensis*, *A*. *pseudopiperis*, and *A*. *pseudotubingensis* were able to solubilize different insoluble minerals (cobalt, copper, ferric, magnesium, manganese, phosphorus, zinc-containing minerals, feldspar, and kaolin), while the solubilization process indicated the very different activities of the different minerals. Notably, phosphorus is the most important key element in terms of the nutrition of plants and is found abundantly in soil; however, it is mostly present in insoluble forms. Therefore, several previous studies have focused on mineral-solubilizing fungi for the solubilization of insoluble phosphorus minerals (Sharma et al., 2013; Alori et al., 2017; Mendoza-Arroyo et al., 2020). The phosphate solubilization indices on the agar plates of *A. chiangmaiensis* (SDBR-CMUI4 and SDBR-CMU15), *A*. *pseudopiperis* (SDBR-CMUI1 and SDBR-CMUI7), and *A*. *pseudotubingensis* (SDBR-CMUO2, SDBR-CMUO8, and SDBR-CMU20) in this study, along with the relevant SI values, were characterized as the medium solubilization activity (SI values between 1.0 and 2.0). These results were similar to previous studies, which found that *A. nigri*, *A. awamori*, and *A. japonicus* in *Aspergillus* section *Nigri* isolated from the soil displayed a medium degree of activity in phosphate solubilization (Alam et al., 2002; Singh et al., 2011; Yadav et al., 2011, 2020; Mahamuni et al., 2012; Das et al., 2013; Verma and Ekka, 2015; Ceci et al., 2018; Islam et al., 2019). Moreover, a high degree of activity of phosphate solubilization (IS value > 2.0) was obtained from *Aspergillus niger* strain-1 (Singh et al., 2011) and strain No. 1 (El-Ghandour et al., 2018). Additionally, the phosphate solubilization was found in some *Aspergillus* species categorized in other *Aspergillus* sections, e.g., *Aspergillus clavatus*, *Aspergillus flavus*, *Aspergillus floccosus*, *Aspergillus fumigatus*, *Aspergillus niveus*, and *Aspergillus versicolor* (Alam et al., 2002; Singh et al., 2011; Yadav et al., 2011, 2020; Mahamuni et al., 2012; Das et al., 2013; Verma and Ekka, 2015; Ceci et al., 2018; Islam et al., 2019). Furthermore, the solubilization abilities of cobalt, ferric, manganese, phosphorus, zinc-containing minerals, and feldspar in *A. niger* and *A. tubingensis* isolated from soil have been reported (Sayer et al., 1995; Gaind, 2016; Kasana et al., 2017; Bakri, 2019). Previous studies have demonstrated that several mineral-solubilizing fungi could solubilize inorganic minerals into available forms through mechanisms that mainly involve the production of organic acids (e.g., citric, fumaric, gluconic, malic, succinic, tartaric, and oxalic acids), hydrolytic enzymes (phytase and phosphatases of phosphorus), and metal-chelating substances (Pawar and Thaker, 2009; Jain et al., 2012b, 2014; Acevedo et al., 2014; Xiao et al., 2015; Zhao and Zhang, 2015).

In this present study, the inoculation of the most effective mineral-solubilizing fungi (*A. chiangmaiensis* SDBR-CMUI4, *A. pseudopiperis* SDBR-CMUI1, and *A. pseudotubingensis* SDBR-CMUO2) could effectively increase plant growth in both Arabidopsis and onion plants. Moreover, Arabidopsis and onion plants that were inoculated with each mineral-solubilizing fungus and those that were supplemented with insoluble mineral phosphate efficiently enhanced plant growth in laboratory and greenhouse experiments, respectively. These results are supported by the findings of previous studies that reported that mineral-solubilizing fungi including *Aspergillus* spp. were able to solubilize insoluble mineral phosphate in soil, while increasing the available amount of phosphorus in the soil for plant growth and productivity (Khan et al., 2010; Singh and Reddy, 2011; Sharma et al., 2013; de Oliveira Mendes et al., 2017). For example, Zhao et al. (2018) and Saxena et al. (2016) found that the inoculation of phosphate-solubilizing fungus, *A. tubingensis* QF05, and *A. niger* K7 could significantly improve the seed germination and plant growth of tomato plants (*Solanum lycopersicum*) in greenhouse experiments, as well as in soybean plants (*Glycine max*) in field experiments, respectively. The inoculation of phosphate-solubilizing fungi, e.g., *Aspergillus aculeatus* P93, *Aspergillus awamori* S19, *A. niger* PSF-7, and *A. tubingensis* PSF-4, used in the supplementation of insoluble mineral phosphate could increase the plant growth and productivity of maize (*Zea mays*), mungbeans (*Vigna radiata*), and wheat (*Triticum aestivum*) in greenhouse experiments and in the field (Jain et al., 2010; Kaur and Reddy, 2016; Yin et al., 2017). Moreover, experiments conducted by Hongmei et al. (2018) indicated that the inoculation of *A. japonicus* M1 could significantly enhance the plant growth and yields of corn in greenhouse experiments and peanut plants in the field due to an increase in the amount of available phosphorus in the soil. Our results indicated that the inoculation of selected fungal strains also increased the chlorophyll content and cellular inorganic phosphate in both Arabidopsis and onion plants along with the relevant chemical constituents (total soluble solids, titratable acidity, and quercetin content) in onion bulbs. Similarly, an experiment conducted by Lubna et al. (2018) found that an inoculation of phosphate-solubilizing fungus, *A. niger* CSR3, significantly enhanced the chlorophyll content and chemical constituents (flavonoid, total phenolic content, and total sugar) of maize. The inoculation of mineral-solubilizing fungi, e.g., *A. awamori* S19, *A. tubingensis* PSF-4, *A. niger* K7, and *A. niger* PSF-7, could improve phosphorus content in maize, mung beans, soybeans, and wheat (Jain et al., 2010; Kaur and Reddy, 2016; Saxena et al., 2016). Moreover, Abdel-Motaal et al. (2020) and Wu et al. (2020) found that the chlorophyll content and secondary metabolite contents of tomato and false indigo bush (*Amorpha fruticosa*) plants were improved after inoculation of the phosphate-solubilizing fungal strains of *A. flavus* and *A. niger*, respectively.

## Conclusion

Soil possesses a diverse fungal population including mineral-solubilizing fungi that can increase the availability of nutrients in the soil. In this study, seven strains of mineral-solubilizing fungi were obtained from the soil taken from a longan orchard located in Northern Thailand. All of the strains were identified as three new species, namely, *A. chiangmaiensis*, *A*. *pseudopiperis*, and *A*. *pseudotubingensis* based on multilocus phylogenetic and phenotypic (morphology and extrolite profile) data. All strains displayed the ability to solubilize insoluble minerals. Inoculation was most effectively achieved in the fungal strains of *A. chiangmaiensis* SDBR-CMUI4, *A. pseudopiperis* SDBR-CMUI1, and *A. pseudotubingensis* SDBR-CMUO2, which then increased plant growth in both Arabidopsis and onion plants. All selected fungal strains did not serve as pathogens on Arabidopsis and onion plants. Moreover, growth enhancement, chlorophyll content, and cellular inorganic phosphate content in both Arabidopsis and onion plants, along with yields of onions and relevant chemical constituents (total soluble solids, titratable acidity, and quercetin content) in onion bulbs were significantly improved when they were inoculated with mineral-solubilizing fungi under conditions supplemented with the insoluble mineral phosphate. Therefore, it is possible that the three new mineral-solubilizing fungi could be used as biofertilizers for plant growth promotion. Further studies involving the mechanisms for their insoluble mineral solubilization (chelating compounds, enzymes, and organic acid production) and plant growth promotion properties (phytohormones and plant growth promotion substances) will need to be conducted. In order to fulfill our understanding of the potential applications of each fungal strain, toxicity assay in the laboratory, clinical tests, and studies of pathogenicity will be required. Additionally, these mineral-solubilizing fungi will need to be applied to other plants, while field trials will also need to be conducted in the future.

## Data Availability Statement

The DNA sequences generated in this study have been deposited in GenBank under the accession numbers; *cam* (MK457199, MW602897, MK457193, MW602902, MK457205, MW602907, and MW602912), *benA* (MK457200, MW602898, MK457194, MW602903, MK457206, MW602908, and MW602913), *rpb2* (MK457202, MW602899, MK457196, MW602904, MK457208, MW602909, and MW602914), *tef1* (MK457203, MW602900, MK457197, MW602905, MK457209, MW602910, and MW602915), and *act* (MK457201, MW602901, MK457195, MW602906, MK457207, MW602911, and MW602916). The sequence alignment was deposited in TreeBASE under the study ID 27054. New fungal taxa were deposited in MycoBank under number MB830887, MB830888, and MB830889.

## Author Contributions

SK, NS, JK, and SL contributed to the conception and design of the study. SK, NS, and JF performed the experiments. SK, NS, JK, JF, and KM analyzed the data and conducted data curation. WN and KM supported the place and provided resources. SK, NS, and JK wrote the original manuscript. SL supervised the study. All authors have read, revised, and approved the final manuscript.

## Conflict of Interest

The authors declare that the research was conducted in the absence of any commercial or financial relationships that could be construed as a potential conflict of interest.

## Publisher’s Note

All claims expressed in this article are solely those of the authors and do not necessarily represent those of their affiliated organizations, or those of the publisher, the editors and the reviewers. Any product that may be evaluated in this article, or claim that may be made by its manufacturer, is not guaranteed or endorsed by the publisher.

## Footnotes

1
http://blast.ncbi.nlm.nih.gov

## Supplementary Material

Supplementary Figure 1 HPLC chromatograms of onion bulb for quantification of quercetin. **(A)** quercetin standard, **(B)** extract of onion in T1, **(C)** extract of onion in T2, **(D)** extract of onion in T3, **(E)** extract of onion in T4, **(F)** extract of onion in T5, **(G)** extract of onion in T6, **(H)** extract of onion in T7, **(I)** extract of onion in T8.

Supplementary Table 1 Details of sequences used for phylogenetic analysis.

Supplementary Table 2 Sclerotium formation by biseriate species of *Aspergillus* section *Nigri*.

Supplementary Table 3 Microscopic characteristics of biseriate species of *Aspergillus* section *Nigri*.

Supplementary Table 4 Extrolite production by biseriate species of *Aspergillus* section *Nigri*.
