# Supplementary material for: Growth Enhancement of Arabidopsis (Arabidopsis thaliana) and Onion (Allium cepa) With Inoculation of Three Newly Identified Mineral-Solubilizing Fungi in the Genus Aspergillus Section Nigri
Source: Front Microbiol. 2021 Aug 12;12:705896. doi: 10.3389/fmicb.2021.705896 (PMC8397495; doi:10.3389/fmicb.2021.705896)
Supplement: Supplementary Figure 1 — HPLC chromatograms of onion bulb for quantification of quercetin. (A) quercetin standard, (B) extract of onion in T1, (C) extract of onion in T2, (D) extract of onion in T3, (E) extract of onion in T4, (F) extract of onion in T5, (G) extract of onion in T6, (H) extract of onion in T7, (I) extract of onion in T8. [file Data_Sheet_1.docx]

**Supplementary TABLE S1.** Details of sequences used for phylogenetic analysis.

| ***Aspergillus* species** | **Strain/isolate** | **GenBank accession number** | | | | |
| --- | --- | --- | --- | --- | --- | --- |
|  |  | ***cam*** | ***benA*** | ***rpb2*** | ***tef1*** | ***act*** |
| *A*. *aculeatinus*^a,b,c^ | CBS 121060^T^ | EU159241 | EU159220 | HF559233 | HF559230 | XM025643717 |
| *A*. *aculeatinus*^b^ | ITEM 13553 | HE984422 | HE984407 | HE984359 | HE984385 | – |
| *A*. *aculeatus*^b,d,e^ | CBS 172.66^T^ | EF661148 | HE577806 | EF661046 | HE984381 | – |
| *A*. *aculeatus*^b^ | ITEM 14807 | HE984424 | HE984409 | HE984372 | HE984398 | – |
| *A*. *brasiliensis*^d,f,g,h^ | CBS 101740^T^ | AM295175 | AY820006 | EF661063 | FN665411 | – |
| *A*. *brasiliensis*^d^ | NRRL 26651 | EF661160 | EF661094 | EF661064 | – | – |
| *A*. *runneoviolaceus*^b,c,d^ | CBS 621.78^T^ | EF661147 | EF661105 | EF661045 | HE984384 | XM025589210 |
| *A*. *carbonarius*^d,f,h,i^ | CBS 111.26^T^ | EF661167 | GU296700 | EF661068 | FN665412 | – |
| *A*. *carbonarius*^d^ | NRRL 4849 | EF661168 | EF661100 | EF661069 | – | – |
| ***A*.** ***chiangmaiensis*^j^** | **SDBR-CMUI4^T^** | **MK457199** | **MK457200** | **MK457202** | **MK457203** | **MK457201** |
| ***A*. *chiangmaiensis*^j^** | **SDBR-CMU15** | **MW602897** | **MW602898** | **MW602899** | **MW602900** | **MW602901** |
| *A*. *costaricaensis*^b,c,f,k^ | CBS 115574^T^ | FN594545 | FJ629277 | HE984361 | FN665409 | XM025685792 |
| *A*. *ellipticus*^b,d,l^ | CBS 482.65^T^ | AM117809 | FJ629279 | EF661051 | HE984386 | – |
| *A*. *ellipticus*^m^ | IHEM 5805 | MH645032 | MH614571 | – | – | – |
| *A*. *eucalypticola*^c,k^ | CBS 122712^T^ | EU482433 | EU482435 | XM025529966 | XM025532307 | XM025535165 |
| *A*. *eucalypticola*^n^ | NRRL 62632 | KC796376 | KC796360 | KC796428 | – | – |
| *A*. *fijiensis*^b,o^ | CBS 119.49 ^T^ | FJ491701 | FJ491689 | HE984362 | HE984387 | – |
| *A*. *fijiensis*^b^ | ITEM 14784 | HE984426 | HE984411 | HE984374 | HE984400 | – |
| *A*. *floridensis*^b^ | NRRL 62478^T^ | HE984429 | HE984412 | HE984376 | HE984403 | – |
| *A*. *floridensis*^b^ | CCF 4046 | HE578097 | HE577817 | – | – | – |
| *A*. *heteromorphus*^b,c,d,k^ | CBS 117.55 ^T^ | AM421461 | FJ629284 | EF661050 | HE984388 | XM025543164 |
| *A*. *heteromorphus*^m^ | IHEM 18645 | MH645030 | MH614573 | – | – | – |
| *A*. *homomorphus*^b,c,g,k^ | CBS 101889^T^ | FN594549 | AY820015 | HE984365 | HE984390 | XM025695935 |
| *A*. *hydei*^p^ | KUMCC 18-0196^T^ | MT178247 | MT161679 | MT384370 | – | – |
| *A*. *ibericus*^b,c,d,f,l^ | ITEM 4776^T^ | AJ971805 | AM419748 | EF661065 | HE984391 | XM025720832 |
| *A*. *ibericus*^d^ | NRRL 35645 | EF661164 | EF661101 | – | – | – |
| *A*. *indologenus*^b,k,q^ | CBS 114.80^T^ | AM419750 | AY585539 | HE984366 | HE984392 | – |
| *A*. *japonicus*^b,c,d,q,r^ | CBS 114.51^T^ | AJ964875 | AY585542 | EF661047 | HE984393 | XM025672389 |
| *A*. *japonicus*^b,d^ | NRRL 35494 | EU021690 | EU021665 | EU021639 | HE984394 | - |
| *A*. *lacticoffeatus*^b,c,g,h^ | CBS 101883^T^ | FN594552 | AY819998 | HE984367 | FN665406 | XM025600201 |
| *A*. *labruscus*^s^ | ITAL 22.223^T^ | KT986008 | KT986014 | – | – | – |
| *A*. *labruscus*^s^ | ITAL 22.227 | KT986009 | KT986015 | – | – | – |
| *A*. *luchuensis*^t^ | CBS 205.80^T^ | JX500071 | JX500062 | LC179910 | – | – |
| *A*. *neoniger*^c,n^ | CBS 115656^T^ | KC796377 | KC796361 | KC796429 | XM025622602 | XM025627117 |
| *A*. *neoniger*^c^ | CMV005B6 | MK451453 | MK451031 | MK450787 | – | – |
| *A*. *niger*^d,h^ | CBS 554.65^T^ | EF661154 | EF661089 | EF661058 | FN665404 | – |
| *A*. *piperis*^c,g,n^ | CBS 112811^T^ | FN594554 | AY820013 | KC796427 | XM025660682 | XM025660053 |
| *A*. *piperis*^c^ | CMV011A9 | MK451493 | MK451187 | MK450798 | – | – |
| ***A*. *pseudopiperis*^j^** | **SDBR-CMUI1^T^** | **MK457193** | **MK457194** | **MK457196** | **MK457197** | **MK457195** |
| ***A*. *pseudopiperis*^j^** | **SDBR-CMUI7** | **MW602902** | **MW602903** | **MW602904** | **MW602905** | **MW602906** |
| ***A*. *pseudotubingensis*^j^** | **SDBR-CMUO2^T^** | **MK457205** | **MK457206** | **MK457208** | **MK457209** | **MK457207** |
| ***A*. *pseudotubingensis*^j^** | **SDBR-CMUO8** | **MW602907** | **MW602908** | **MW602909** | **MW602910** | **MW602911** |
| ***A*. *pseudotubingensis*^j^** | **SDBR-CMU20** | **MW602912** | **MW602913** | **MW602914** | **MW602915** | **MW602916** |
| *A*. *saccharolyticus*^b,u^ | CBS 127449 ^T^ | HM853554 | HM853553 | HF559235 | HF559232 | – |
| *A*. *sclerotiicarbonarius*^a^ | CBS 121057^T^ | EU159235 | EU159229 | – | – | – |
| *A*. *sclerotiicarbonarius*^a^ | CBS 121851 | EU159239 | EU159230 | – | – | – |
| *A*. *sclerotioniger*^a,b,c,g^ | CBS 115572^T^ | EU163271 | AY819996 | HE984369 | HE984396 | XM025606480 |
| *A*. *serratalhadensis*^v^ | URM 91189^T^ | LT993223 | LT993222 | LT995971 | – | – |
| *A*. *trinidadensis*^b^ | NRRL 62479^T^ | HE984434 | HE984420 | HE984379 | HE984406 | – |
| *A*. *trinidadensis*^b^ | NRRL 62480 | HE984425 | HE984410 | HE984373 | HE984399 | – |
| *A*. *tubingensis*^d,h^ | CBS 134.48^T^ | EF661151 | EF661086 | EF661055 | FN665407 | – |
| *A*. *tubingensis*^w^ | PW3161 | LC000560 | LC000547 | LC000573 | LC382162 | – |
| *A*. *uvarum*^b,c,x^ | CBS 127591^T^ | AM745755 | AM745751 | HE984370 | HE984397 | XM025637406 |
| *A*. *uvarum*^b^ | ITEM 14819 | HE984435 | HE984421 | HE984380 | – | – |
| *A*. *vadensis*^a,b,c,h,q^ | CBS 113365^T^ | EU163269 | AY585531 | HE984371 | FN665408 | XM025707316 |
| *A*. *vadensis*^m^ | IHEM 26351 | MH644878 | MH614547 | – | – | – |
| *A*. *vinaceus*^y^ | ITAL 47.456^T^ | MN583580 | MN583579 | MN583581 | – | – |
| *A*. *welwitschiae*^k,s^ | CBS 139.54^T^ | KC480196 | FJ629291 | – | – | – |
| *A*. *welwitschiae*^x^ | PW3050 | LC000559 | LC000546 | LC000572 | LC425550 | – |
| *A*. *fischeri*^c,d^ | CBS 544.65^T^ | EF669865 | EF669796 | EF669724 | XM001265027 | XM001257680 |
| *A*. *novofumigatus*^c,z^ | CBS 117520^T^ | DQ094893 | DQ094886 | LC367690 | XM024825699 | XM024825612 |

^a^Noonim et al. (2008), ^b^Jurjević et al. (2012), ^c^Unpublished, ^d^Peterson (2008), ^e^Hubka and Kolarik (2012), ^f^Varga et al. (2007), ^g^Samson et al. (2004), ^h^Perrone et al. (2011), ^i^Andersen et al. (2011), ^j^This study, ^k^Samson et al. (2014), ^l^Serra et al. (2006), ^m^D'hooge et al. (2018), ^n^Horn et al. (2013), ^o^Varga et al. (2011), ^p^Doilom et al. (2020), ^q^de Vries et al. (2005), ^r^Perrone et al. (2006), ^s^Fungaro et al. (2017), ^t^Hong et al. (2013), ^u^Sørensen et al. (2011), ^v^Crous et al. (2018), ^w^Tsang et al. (2016), ^x^Perrone et al. (2008), ^y^da Silva et al. (2020), ^z^Hong et al. (2005). Superscript T in the strain/isolate column indicates a type species.

**Supplementary TABLE S2.** Sclerotium formation by biseriate species of *Aspergillus* section *Nigri*.

| *Aspergillus* species | Size (µm) | Shape | Color | Culture medium |
| --- | --- | --- | --- | --- |
| *A. chiangmaiensis*^a^ | 390–1375 × 390–1085 | Globose to ellipsoidal | White | CZA, PDA |
| *A. costaricaensis*^b^ | 1200–1800 | Subglobose to ellipsoidal | Pink to grayish yellow | CYA, MEA |
| *A. ellipticus*^c^ | 500–1500 | Discoid form | Dull yellowish to brown | CZA |
| *A. heteromorphus*^d^ | 100–500 | Globose or subglobose | Brown | CYA, MEA |
| *A. piperis*^b^ | 1000–1700 | Subglobose to ellipsoidal | Yellow to pink brown | CYA, MEA, OA YES |
| *A. pseudopiperis*^a^ | 200–985 × 185–765 | Globose to ellipsoidal | Light yellow to pinkish orange | CYA, CZA, MEA, OA, PDA, YES |
| *A. sclerotiicarbonarius*^e^ | 800–1200 | Globose to ellipsoidal and irregular | Yellow to orange to red brown | CYA, CZA, MEA, OA, YES |
| *A. sclerotioniger*^b^ | 1000–1600 | Globose | Yellow to orange to red brown | CYA, MEA |
| *A. tubingensis*^f^ | 500–1600 × 500–1350 | Globose to ellipsoidal | Cream to pinkish tan | CZA, MEA |
| *A. vinaceus*^g^ | 900–1500 | Ovoid | White to cream, | CYA, MEA, YESA |

^a^This study, ^b^Samson et al. (2004), ^c^Samson et al. (2007), ^d^Eltem et al. (2004), ^e^Noonim et al. (2008), ^f^Horn et al. (2013), ^g^da Silva et al. (2020).

**Supplementary TABLE S3.** Microscopic characteristics of biseriate species of *Aspergillus* section *Nigri*.

| ***Aspergillus* species** | **Conidial heads**  **(µm)** | **Stipes**  **(µm)** | **Vesicles**  **(µm)** | **Metulae**  **(µm)** | **Phialides**  **(µm)** | **Conidia (µm)** | **Conidia shape** |
| --- | --- | --- | --- | --- | --- | --- | --- |
| *A. brasilliensis*^a^ | NR | 700–1700 × 8–13 | 30–45 | 22–30 × 3–6 | 7–9 × 3–4 | 3.5–4.8 | Subglobose, echinulate |
| *A. carbonarius*^b^ | NR | NR | 49–85 | NR | NR | 7–10 | Globular, wrinkled |
| *A. chiangmaiensis*^c^ | 60–225 | 370–1430 × 10–15 | 35–68 | 14–25 × 3–5 | 8–12 × 2–3 | 3–4.5 | Globose to subglobose, echinulate |
| *A. costaricaensis*^d^ | NR | 1000–1700 × 13–20 | 45–70 | 30–60 × 3–11 | 7–9.5 × 3–5 | 3.5–4.3 | Globose to subglobose, smooth to distinct rough walled |
| *A. ellipticus*^e^ | 400–700 | 5000–8000 × 12–20 | 70–100 | 20–40 × 5.5–7.5 | 9–11 × 3.5–4.5 | 3.3–5.5 | Globose, echinulate with spines discrete |
| *A. eucalypticola*^f^ | NR | 8–14 | 30–55 | NR | NR | 2.5–3.5 | Globose, smooth walled to coarsely roughened |
| *A. heteromorphus^g^* | 180–200 | 800–900 × 8.0–12 | 35–45 | 10–12 × 3.5–4 | 6–8 × 2.5–3 | 3–3.5 | Verruculose |
| *A. homomorphus*^h^ | 80–180 | 400–875 × 9–15 | 27–50 | 7–8 (long) | 9–15 × 4–5 | 5–8 | Globose to subglobose, conspicuously spiny to echinate |
| *A. ibericus*^i^ | 500–600 | 1200–2000 × 14–20 | 50–60 | 30–40 × 5.0–7.5 | 8–10 × 6–7 | 5–7 | Globose to subglobose, conspicuously verruculose with spines projecting |
| *A. lacticoffeatus*^d^ | NR | 300–1200 × 10–15 | 45–60 | 12–25 × 3–6 | 7–10 × 3–4 | 3.4–4.1 | Subglobose, usually smooth to very finely roughenced |
| *A. luchuensis*^j^ | NR | 1500 × 10–13 | 20–40 | 17–26.1×  4.5–8.1 | 5.6–8.4 × 3.5–4.9 | 3.5–4.5 | Globose, smooth |
| *A. neoniger*^f^ | NR | 8–12 | 30–50 | NR | NR | 3.5–5 | Globose, coarsely roughened to echinulate |
| *A. niger*^k^ | 750–850 | 1500–3000 | 45–75 | NR | NR | 2.5–10 | Spherical or subspherical,  with prominent warts and ridges |
| *A. piperis*^d^ | NR | 400–3000 × 12–15 | 45–50 | 25–30 × 3–6 | 6–7.5 × 3–4 | 2.8–3.6 | Subglobose to broadly ellipsoidal, smooth when young to very rough with irregular bars/striations |
| *A. pseudopiperis*^c^ | 50–245 | 200–2125 × 10–18 | 15–55 | 15–39 × 3–7 | 9–14 × 3–4 | 3–5 | Globose to subglobose, ridged |
| *A. pseudotubingensis*^c^ | 50–255 | 740–3060 × 10–18 | 20–70 | 14–46 × 3–6 | 9–14 × 3–4 | 3–6 | Globose to subglobose, finely spinose |
| *A. sclerotiicarbonarius*^l^ | NR | 1400–2400 × 15–23 | 55–75 | 32–39 × 2–4 | 4–14 × 4–7 | 5.8–8 | Subglobose, conspicuously spiny to verruculose |
| *A. sclerotioniger*^d^ | NR | 500–800 × 14–16 | 35–45 | 8–14 × 4–6 | 6.5–9.5 × 3–5 | 4.9–6 | Subglobose, smooth to verruculose |
| *A. tubingensis*^m^ | ≤700 | 550–2300 × 10–18 | 35–60 | 5–8 × 3–4.8 | 5.5–10 × 3–4.7 | 3.2–4.8 | Globose to subglobose, tuberculate to aculeate with ridges |
| *A. vadensis*^n^ | NR | ≤150 × 6–15 | 25–35 | NR | NR | 3–4 | Globose, rough walled to finely echinulate |
| *A. vinaceus*^o^ | up to 200 | 1300–1800 × 16–21 | 63–75 | 20–40 | 15–27 | 3–5.5 | Globose to sub-globose, finely roughened to echinulate |
| *A. welwitschiae*^j^ | NR | NR | 45–85 | NR | NR | 3.5–5.5 | Globose, finely to distinctly  roughened |

^a^Varga et al. (2007), ^b^Silva et al. (2011), ^c^This study, ^d^Samson et al. (2004), ^e^Raper and Fennell (1965), ^f^Varga et al. (2011), ^g^Eltem et al. (2004), ^h^Hussein et al. (2017), ^i^Serra et al. (2006), ^j^Hong et al. (2013), ^k^Gugnani (2003), ^l^Noonim et al. (2008), ^m^Horn et al. (2013), ^n^de Vries et al. (2005), ^o^da Silva et al. (2020). “NR” = not reported.

**Supplementary TABLE S4.** Extrolite production by biseriate species of *Aspergillus* section *Nigri*.

| ***Aspergillus* species** | **Extrolites produced** |
| --- | --- |
| *A. brasilliensis*^a^ | Aurasperone B, brasenol, dihydrocarolic acid, malformins, pyrophen, tensidol A and B, 14-epi-14-hydroxy-10,23-dihydro-24,25-dehydroaflavinine, 10,23-dihydro-24,25-dehydroaflavinine |
| *A. carbonarius*^b^ | Aurasperone B, brasenol, ochratoxins A, B, α, and β, pyranonigrin A |
| *A. chiangmaiensis*^c^ | Desertorin C, pyrophen, secalonic acid D, a unique ustilaginoidin-like compound |
| *A. costaricaensis*^d^ | Aurasperone B, funalenone, pyranonigrin A, 14-epi-14-hydroxy-10,23-dihydro-24,25-dehydroaflavinine, 10,23-dihydro-24,25- dehydroaflavinine |
| *A. ellipticus*^b^ | Austdiol, candidusins, cf. xanthoascin, terpenyllin |
| *A. eucalypticola*^e^ | Aurasperone B, funalenone, naphtho-γ-pyrones, pyranonigrin A |
| *A. heteromorphus*^b^ | Many highly unique extrolites are produced, not yet structure elucidated |
| *A. homomorphus*^b^ | Dehydrocarolic acid, homomorphosins, secalonic acid D and F |
| *A. ibericus*^f^ | An aflavinine |
| *A. lacticoffeatus*^d^ | Ochratoxin A, ochratoxin B, pyranonigrin A, orlandin, kotanin |
| *A. luchuensis*^f,g^ | Antafumicins and/or luchuensin, atromentin, funalenone, pyranonigrin A, occasionally tensidol B, 10,23-dihydro-24,25-dehydroaflavinine |
| *A. neoniger*^e,f^ | Aurasperone B, pyranonigrin A, 10,23-dihydro-24,25-dehydroaflavinine |
| *A. niger*^b,f^ | Aurasperone B, fumonisin B_2_, B_4_, and B_6_, funalenone, malformins, nigragillin, ochratoxin A (only some strains), pyranonigrin A, tensidol A and B, 10,23-dihydro-24,25-dehydroaflavinine |
| *A. piperis*^d^ | Aurasperone B, 14-epi-14-hydroxy-10,23-dihydro-24,25-dehydroaflavinine, 10,23-dihydro-24,25-dehydroaflavinine |
| *A. pseudopiperis*^c^ | Aflavinines, an emindole, rotiorin, a unique ustilaginoidin-like compound |
| *A. pseudotubingensis*^c^ | Brasenol, tensidol C |
| *A. sclerotiicarbonarius*^f,h^ | Aurasperone B, paspa, pyranonigrin A, three unique indol-alkaloids |
| *A. sclerotioniger*^d^ | Aurasperone B, corymbiferan lactones, funalenone, ochratoxins A and B, pyranonigrin A |
| *A. tubingensis*^b,f,i^ | Asperazine, aspernomine, aurasperone B, dehydrotubingensin A and B, funalenone, malformins, nigragillin, pyranonigrin A, tensidol A and B, tubingensin A and B,14-hydroxy-10,23-dihydro-24,25-dehydroaflavinine, 14-epi-14-hydroxy-10,23-dihydro-24,25-dehydroaflavinine, 10,23-dihydro-24,25-dehydroaflavinine, 10,23-dihydro-24,25-dehydro-21-oxo-aflavinine |
| *A. vadensis*^j^ | Aurasperone B, asperazine, kotanins, nigragillin |
| *A. vinaceus*^k^ | Acetyl-leucomelone, asperazine, aurasperone B, C, and F, fonsecin B, funalenones, HUTI, malformin A1 and C, nigragillin, pyranonigrin A, SURI, tensidol B |
| *A. welwitschiae*^g,l^ | Fumonisin B_2_, B_4_ and B_6_, ochratoxins |

^a^Varga et al. (2007), ^b^Samson et al. (2007), ^c^This study, ^d^Samson et al. (2004), ^e^Varga et al. (2011), ^f^Frisvad et al. (2014), ^g^Hong et al. (2013), ^h^Noonim et al. (2008), ^i^TePaske et al. (1989), ^j^de Vries et al. (2005), ^k^da Silva et al. (2020), ^l^Frisvad et al. (2011).


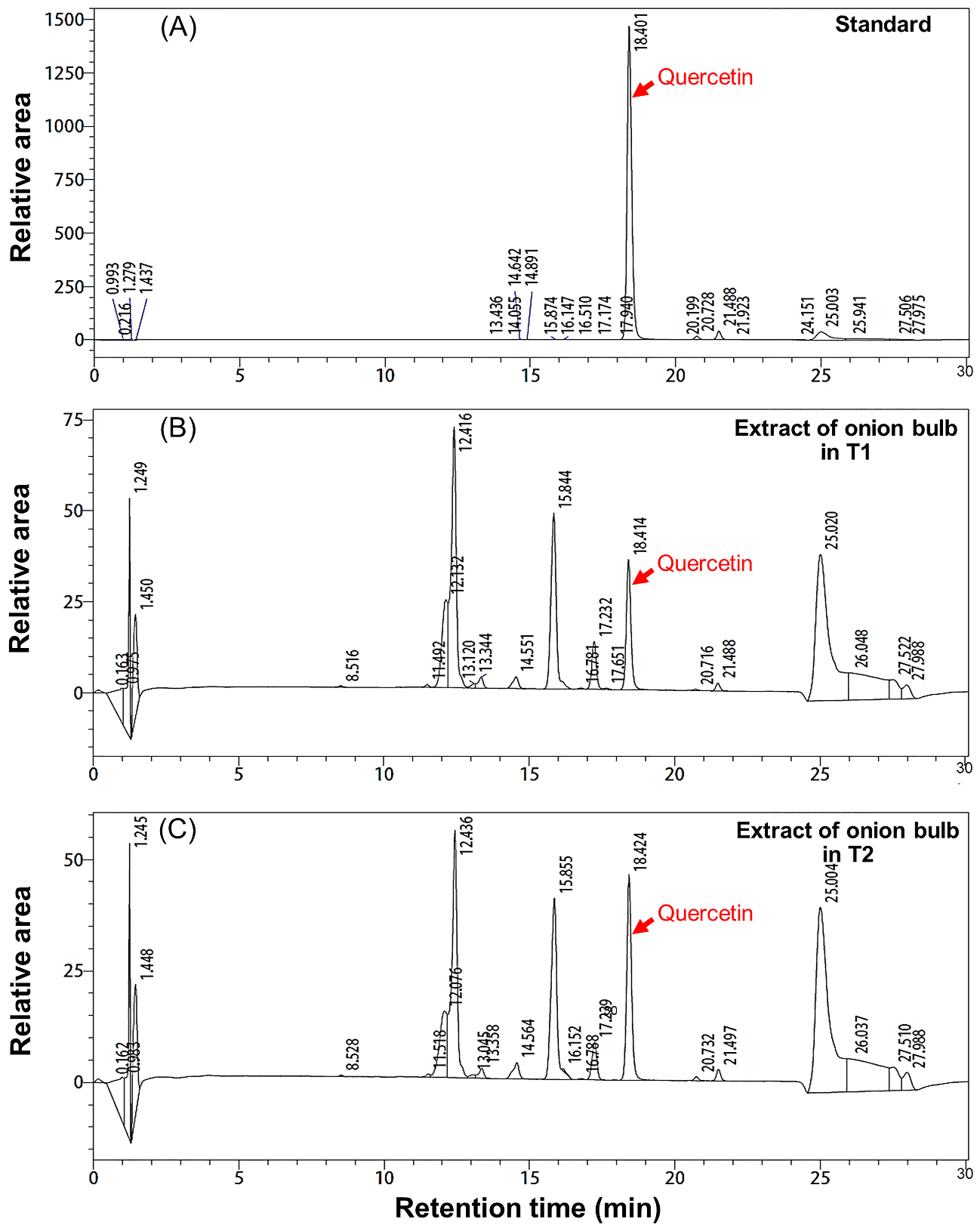


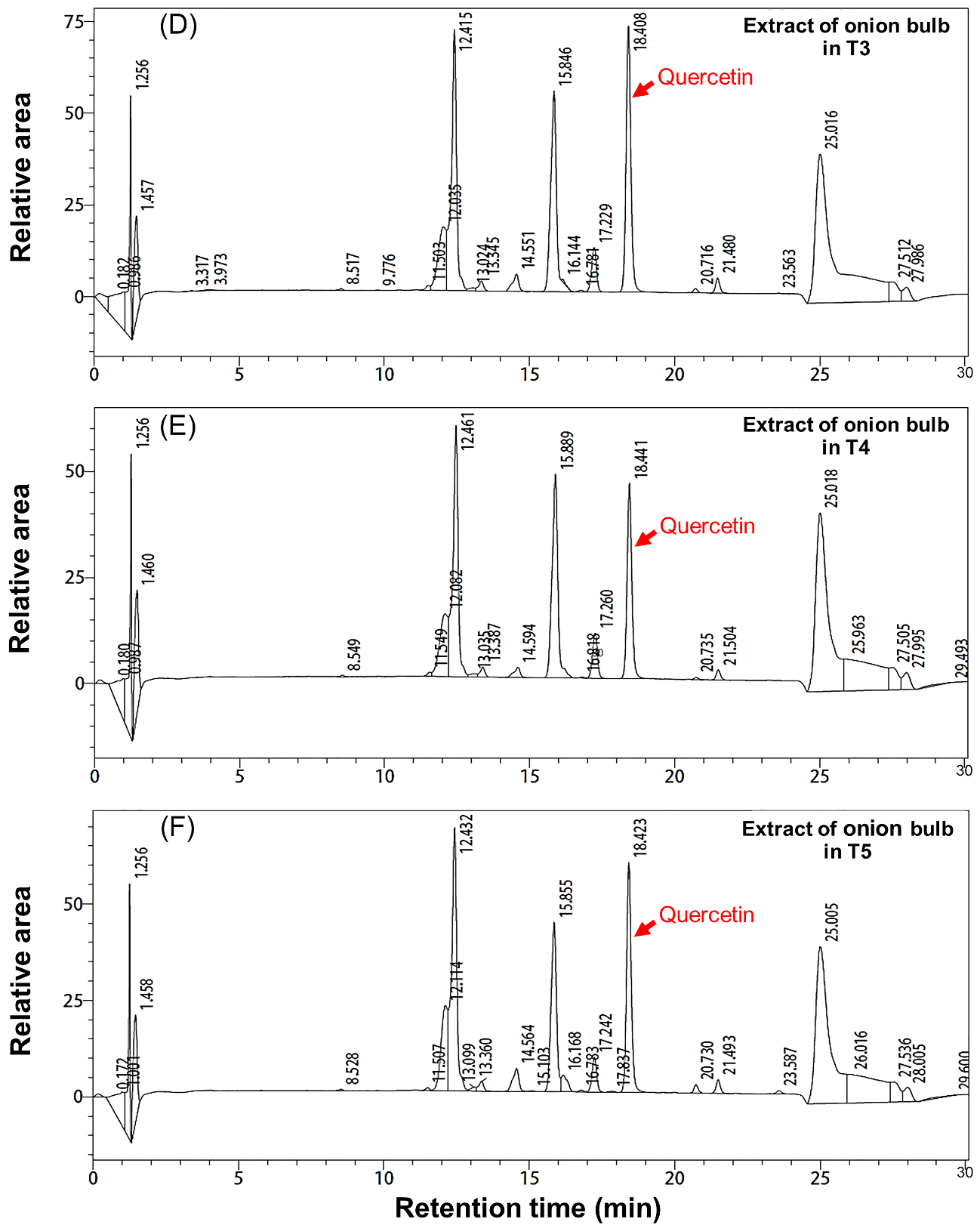


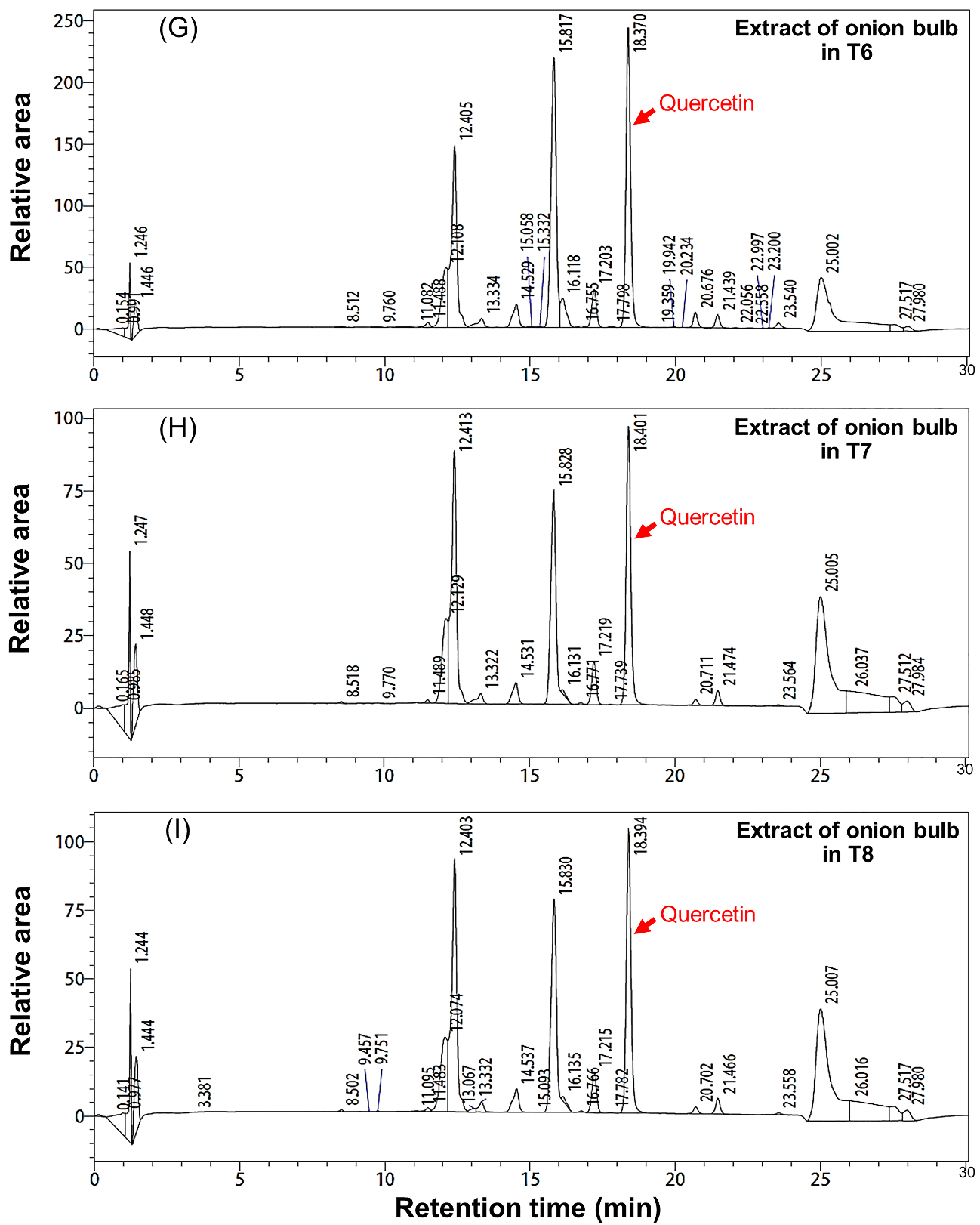


**Supplementary FIGURE S1.**

HPLC chromatograms of onion bulb for quantification of quercetin. **(A)** quercetin standard, **(B)** extract of onion in T1, **(C)** extract of onion in T2, **(D)** extract of onion in T3, **(E)** extract of onion in T4, **(F)** extract of onion in T5, **(G)** extract of onion in T6, **(H)** extract of onion in T7, **(I)** extract of onion in T8.

**REFERENCES**

Andersen, M. R., Salazar, M. P., Schaap, P. J., van de Vondervoort, P. J., Culley, D., Thykaer, J.,
 et al. (2011). Comparative genomics of citric-acid-producing *Aspergillus niger* ATCC
 1015 versus enzyme-producing CBS 513.88. *Genome Res*. 21, 885–897. doi:
 10.1101/gr.112169.110

Crous, P. W., Wingfield, M. J., Burgess, T. I., Hardy, G. E. St. J., Gené, J., Guarro, J., et al.
 (2018). Fungal planet description sheets: 716–784. *Persoonia* 40, 240–393. doi:
 10.3767/persoonia.2018.40.10

da Silva, J. J., Iamanaka, B. T., Ferranti, L. S., Massi, F. P., Taniwaki, M. H., Puel, O., et al.
 (2020). Diversity within *Aspergillus niger* clade and description of a new
 species: *Aspergillus vinaceus* sp. nov. J. Fungi (Basel) 6:371. doi: 10.3390/jof6040371

de Vries, R. P., Frisvad, J. C., van de Vondervoort, P. J., Burgers, K., Kuijpers, A. F., Samson,
 R. A., et al. (2005). *Aspergillus vadensis*, a new species of the group of black Aspergilli.  *Antonie Van Leeuwenhoek* 87, 195–203. doi: 10.1007/s10482-004-3194-y

D’hooge, E., Becker, P., Stubbe, D., Normand, A. C., Piarroux, R., and Hendrickx, M. (2018).
 Black aspergilli: A remaining challenge in fungal taxonomy?. *Med. Mycol*. 57, 773–780.
 doi: 10.1093/mmy/myy124

Doilom, M., Guo, J. W., Phookamsak, R., Mortimer, P. E., Karunarathna, S. C., Dong, W., et al.
 (2020). Screening of phosphate-solubilizing fungi from air and soil in Yunnan, China:
 four novel species in *Aspergillus*, *Gongronella*, *Penicillium*, and *Talaromyces*. *Front.
 Microbiol*. 11:585215. doi: 10.3389/fmicb.2020.585215

Eltem, R., Aşkun, T., Sarigül, N., Taşkin, E. Ö., and Efendiler, H. (2004). Colonial and
 morphological characteristics of some *Aspergillus* Fr.:Fr. Species isolated from Vineyards
 in Manisa and İzmir Provinces (Turkey). *Turk. J. Bot*. 28, 287–298.

Frisvad, J. C., Larsen, T. O., Thrane, U., Meijer, M., Varga, J., Samson, R. A., et al. (2011).
 Fumonisin and ochratoxin production in industrial *Aspergillus niger* strains. *PloS One*
 6:e23496. doi: 10.1371/journal.pone.0023496

Frisvad, J. C., Petersen, L. M., Lyhne, E. K., and Larsen, T. O. (2014). Formation of sclerotia and
 production of indoloterpenes by *Aspergillus niger* and other species in section *Nigri*. *PloS
 One* 9:e94857. doi: 10.1371/journal.pone.0094857

Fungaro, M. H. P., Ferranti, L. S., Massi, F. P., da Silva, J. J., Sartori, D., Taniwaki, M. H., et al.
 (2017). *Aspergillus labruscus* sp. nov., a new species of *Aspergillus* section *Nigri*
 discovered in Brazil. *Sci. Rep*. 7:6203. doi: 10.1038/s41598-017-06589-y

Gugnani, H. C. (2003). Ecology and taxonomy of pathogenic aspergilli. *Front. Biosci*. 8,
 s346–357. doi: 10.2741/1002

Hong, S. B., Go, S. J., Shin, H. D., Frisvad, J. C., and Samson, R. A. (2005). Polyphasic
 taxonomy of *Aspergillus fumigatus* and related species. *Mycologia* 97, 1316–1329. doi:
 10.3852/mycologia.97.6.1316

Hong, S. B, Lee, M., Kim, D. H., Varga, J., Frisvad, J. C., Perrone, G., et al. (2013). *Aspergillus
 luchuensis*, an industrially important black *Aspergillus* in East Asia. *PloS One* 8:e63769.
 doi: 10.1371/journal.pone.0063769

Horn, B. W., Olarte, R. A., Peterson, S. W., and Carbone, I. (2013). Sexual reproduction in  *Aspergillus tubingensis* from section *Nigri*. *Mycologia* 105, 1153–1163. doi:
 10.3852/13-101

Hubka, V., and Kolarik, M. (2012). Β-tubulin paralogue *tubC* is frequently misidentified as the *benA* gene in *Aspergillus* section *Nigri* taxonomy: primer specificity testing and
 taxonomic consequences. *Persoonia* 29, 1–10. doi: 10.3767/003158512X658123

Hussein, N., Abdel-Hafez, S. I. I., Abdel-Sater, M. A., Ismail, M. A., and AL-Amrey, E. (2017).  *Aspergillus homomorphus*, a first global record from millet grains. *Curr. Res. Environ.
 Appl. Mycol*. 7, 82–89. doi: 10.5943/cream/7/2/4

Jurjević, Ž., Peterson, S. W., Stea, G., Solfrizzo, M., Varga, J., Hubka, V., et al. (2012). Two
 novel species of *Aspergillus* section *Nigri* from indoor air. *IMA Fungus* 3, 159–173. doi:
 10.5598/imafungus.2012.03.02.08

Noonim, P., Mahakarnchanakul, W., Varga, J., Frisvad, J. C., and Samson, R. A. (2008). Two
 novel species of *Aspergillus* section *Nigri* from Thai coffee beans. *Int. J. Syst. Evol.
 Microbiol*. 58, 1727–1734. doi: 10.1099/ijs.0.65694-0

Perrone, G., Mulè, G., Susca, A., Battilani, P., Pietri, A., and Logrieco, A. (2006). Ochratoxin A
 production and amplified fragment length polymorphism analysis of *Aspergillus
 carbonarius*, *Aspergillus tubingensis*, and *Aspergillus niger* strains isolated from grapes in
 Italy. *Appl. Environ. Microbiol*. 72, 680–685. doi: 10.1128/AEM.72.1.680-685.2006

Perrone, G., Varga, J., Susca, A., Frisvad, J. C., and Stea, G. (2008). *Aspergillus uvarum* sp. nov.,
 an uniseriate black *Aspergillus* species isolated from grapes in Europe. *Int. J. Syst. Evol.
 Microbiol*. 58, 1032–1039. doi: 10.1099/ijs.0.65463-0

Perrone, G., Stea, G., Epifani, F., Varga, J., Frisvad, J. C, and Samson, R. (2011). *Aspergillus
 niger* contains the cryptic phylogenetic species *A. awamori*. *Fungal Biol*. 115, 1138–1150.
 doi: 10.1016/j.funbio.2011.07.008

Peterson, S. W. (2008). Phylogenetic analysis of *Aspergillus* species using DNA sequences from
 four loci. *Mycologia* 100, 205–226. doi: 10.3852/mycologia.100.2.205

Raper, K. B., and Fennell, D. I. (1965). The genus *Aspergillus*. Baltimore: Williams & Wilkins.
 686 p.

Samson, R. A., Houbraken, J. A. M. P., Kuijpers, A. F. A., Frank, J. M., and Frisvad, J. C. (2004).
 New ochratoxin A or sclerotium producing species in *Aspergillus* section *Nigri*. *Stud.
 Mycol*. 50, 45–61.

Samson, R. A., Noonim, P., Meijer, M., Houbraken, J., Frisvad, J. C., and Varga, J. (2007).
 Diagnostic tools to identify black aspergilli. *Stud. Mycol*. 59, 129–145. doi:
 10.3114/sim.2007.59.13

Samson, R. A., Visagie, C. M., Houbraken, J., Hong, S. B., Hubka, V., Klaassen, C. H. W., et al.
 (2014). Phylogeny, identification and nomenclature of the genus *Aspergillus*. *Stud.
 Mycol*. 78, 141–173. doi: 10.1016/j.simyco.2014.07.004

Serra, R., Cabañes, F. J., Perrone, G., Castellá, G., Venâncio, A., Mulè, G., et al. (2006).  *Aspergillus ibericus*: a new species of section *Nigri* isolated from grapes. *Mycologia* 98,
 295–306. doi: 10.1080/15572536.2006.11832702

Silva, D. M., Batista, L. R., Rezende, E. F., Fungaro, M. H., Sartori, D., and Alves, E. (2011).
 Identification of fungi of the genus *Aspergillus* section *Nigri* using polyphasic taxonomy.
 Braz. *J. Microbiol*. 42, 761–773. doi: 10.1590/S1517-838220110002000044

Sørensen, A., Lübeck, P. S., Lübeck, M., Nielsen, K. F., Ahring, B. K., Teller, P. J., et al. (2011).  *Aspergillus saccharolyticus* sp. nov., a black *Aspergillus* species isolated in Denmark. *Int.
 J. Syst. Evol. Microbiol*. 61, 3077–3083. doi: 10.1099/ijs.0.029884-0

TePaske, M. R., Gloer, J. B., Wicklow, D. T., and Dowd, P. F. (1989). Three new aflavinines
 from the sclerotia of *aspergillus tubingensis*. *Tetrahedron* 45, 4961–4968. doi:
 10.1016/S0040-4020(01)81077-2

Tsang, C. C., Hui, T. W., Lee, K. C., Chen, J. H., and Ngan, A. H. (2016). Genetic diversity of  *Aspergillus* species isolated from onychomycosis and *Aspergillus hongkongensis* sp. nov.,
 with implications to antifungal susceptibility testing. *Diagn. Microbiol. Infect. Dis*. 84,
 125–134. doi: 10.1016/j.diagmicrobio.2015.10.027

Varga, J., Frisvad, J. C., Kocsubé, S., Brankovics, B., Tóth, B., Szigeti, G., et al. (2011). New and
 revisited species in *Aspergillus* section *Nigri*. *Stud. Mycol*. 69, 1–17. doi:
 10.3114/sim.2011.69.01

Varga, J., Kocsubé, S., Tóth, B., Frisvad, J. C., Perrone, G., Susca, A., et al. (2007). *Aspergillus
 brasiliensis* sp. nov., a biseriate black *Aspergillus* species with world-wide distribution.
 *Int. J. Syst. Evol. Microbiol*. 57, 1925–1932. doi: 10.1099/ijs.0.65021-0
